# Delivering DNA Aptamers Across the Blood–Brain Barrier Reveals Heterogeneous Decreased ATP in Different Brain Regions of Alzheimer’s Disease Mouse Models

**DOI:** 10.1021/acscentsci.4c00563

**Published:** 2024-07-31

**Authors:** Mandira Banik, Aaron P. Ledray, Yuting Wu, Yi Lu

**Affiliations:** † University of Texas at Austin, Department of Chemistry, Austin, Texas 78712, United States

## Abstract

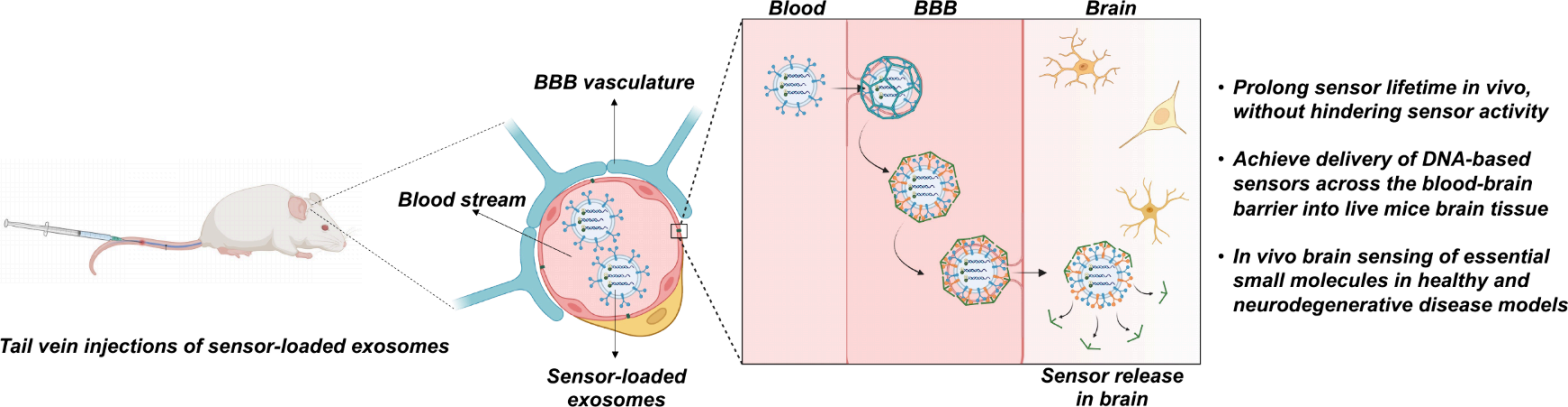

DNA aptamers have been developed as sensors to detect metabolites with high sensitivity, selectivity, and biocompatibility. While they are effective in sensing important targets in the brain, the lack of methods for their efficient delivery across the blood–brain barrier (BBB) has significantly hindered their applications in brain research. To address this issue, we herein report the development of brain cell-derived exosomes as endogenous BBB delivery vehicles to deliver an ATP-responsive aptamer across the BBB of live mice for noninvasive live brain imaging. We found that the system uses endosome recycling to transfer the sensors between the delivered exosomes and native recycling endosomes, resulting in high delivery efficiencies. Using this system, we observed unique signal distributions for ATP across different brain regions, with significant accumulation in the subiculum and cortex in healthy mice. In an Alzheimer’s disease transgenic mouse model, ATP levels decreased in the subiculum and cortex, demonstrating this method’s capability to determine metabolite location and relative abundance with high spatial resolution in vivo. Since DNA aptamers have been obtained for many other targets, the method developed in this work can be applied to deliver sensors across the BBB to image a wide range of other brain-related metabolites.

## Abstract 2

This work describes exosome-mediated delivery of DNA aptamers across the blood−brain barrier in mice to sense the locations and abundances of physiologically relevant metabolites in live brain tissue.

## 1 Introduction

Metabolites, such as ATP, play essential roles in neurobiology by serving as neurotransmitters and maintaining ion gradient flux.^1^ These species often decrease with age, which may result in abnormal cellular signaling that ultimately propagates neurodegenerative diseases.^2^ Therefore, detecting the varying concentrations and cellular locations of these targets in the brain may help with understanding their roles in promoting brain health and preventing neurodegenerative diseases. However, in contrast to progress made in imaging macromolecules, such as nucleic acids and proteins, the selective detection of small molecule metabolites in the brain has been limited. Toward this goal, instrumental methods, such as magnetic resonance and ultrasound, achieve noninvasive whole-brain imaging. However, since the concentrations of these metabolites are relatively low compared to other biomolecules and their distributions are often dynamic, these methods may not provide enough sensitivity, selectivity, or spatial and temporal information.^3,4^ To overcome these limitations, electrochemical sensors have been developed with high sensitivity and selectivity for distinct targets but with only minimal spatial resolution in cells.^5^ To improve sensor resolution, genetically encoded protein biosensors provide subcellular resolution in live cells, with typically fast sensor response time scales. However, these require fine-tuned genetic engineering for different target metabolites, so they have limited generalizability, with often narrow dynamic ranges.^6^ As a result, there are limited techniques to determine the spatial distribution of metabolites in intact live brain tissue, leading to a poor understanding of how the distributions of metabolites and their dynamic changes contribute to brain health and neurodegeneration.

To overcome the above limitations, we are interested in exploring the use of DNA aptamers, which have been shown to be generally applicable to detect a wide variety of metabolites with high selectivity, sensitivity, and biocompatibility. Aptamers can be obtained through iterative rounds of in vitro selection from libraries of up to 10^15^ sequences, with counterselection to remove DNA sequences that can bind structurally similar competing or interfering species, making them highly selective.^7,8^ In addition, these sensors can be readily conjugated to fluorophores, quenchers, and nanoparticles, allowing for generalizable methods to convert the binding of metabolites into fluorescent, photoacoustic, colorimetric, and other sensor outputs with limits of detection reaching the nanomolar or lower level.^9−11^ Furthermore, they exhibit a high degree of biocompatibility, as they can be delivered in micromolar concentrations with a negligible effect on cell viability.^12,13^ As a result, DNA aptamers have seen widespread use in monocultured cells, fixed tissue, and in vivo as sensors and as targeting agents.^12−16^ Moreover, many DNA aptamer sensors for metabolites implicated in brain function, such as ATP, glucose, and dopamine, have already been developed.^17−20^

While DNA aptamer sensors have shown significant potential to be used as sensors in the brain, their application has been limited by the blood–brain barrier (BBB). The BBB is a specialized layer of endothelial cells that preserves brain homeostasis and prevents brain uptake of nonessential small molecules and macromolecules due to the selectivity of tight junctions between the endothelial cells.^21,22^ To overcome this limitation, different BBB-penetrable delivery techniques, such as cell-penetrating peptides, liposomes, microbubbles, and aptamer-based targeting ligands, have been explored.^23−27^ The applications of these methods in vivo are limited due to nuclease degradation, cellular toxicity, and serum interference because these methods use synthetic components that are not native to the brain and/or are not recognizable by BBB transporters.^38^

To overcome the above limitations, we are interested in employing exosomes derived from brain cells as a highly efficient system to deliver DNA aptamer sensors across the BBB and into the brains of live mice for in vivo sensing. Exosomes are membrane-bound extracellular vesicles of ∼100 nm that have been shown to deliver cytosolic components, such as nucleic acids, proteins, and lipids, across the BBB as a means of cell communication and cell waste transfer.^28^ Exosomes derived from brain cells contain proteins and other macromolecules on their surface that can be inherently recognized by the BBB, imparting higher specificity for the BBB and increasing their biocompatibility and delivery efficiency compared with synthetic delivery vehicles.^29−31^ Based on this principle, immune cell-derived exosomes have been used to deliver siRNA, mRNA, microRNA, proteins, and lipids to neurons, microglia, and oligodendrocytes.^32,33^ Given the success of exosomes as natural nucleic acid delivery agents, we hypothesized that brain cell-derived exosomes would be able to deliver the DNA aptamer sensors across the BBB and into the brain.

To test this hypothesis, we applied native exosomes derived from mammalian brain cells for the delivery of aptamers across the BBB for live brain sensing of metabolites. We characterized the exosomes as delivery vehicles for sensor output in monocultured brain endothelial cells, in a static BBB model, and in live mice. We found that exosomes delivered the aptamer sensors across a static BBB model and healthy mice with higher efficiency than nonspecific lipid nanoparticle transfection agents due to exploitation of the recycling endosome pathway. Using this method to deliver an aptamer for adenosine and adenosine-containing molecules, such as ATP, AMP, and ADP, we found these targets have similar distributions overall throughout the healthy brain but significantly higher abundance in the subiculum, an underexplored brain region that coordinates connections between the hippocampus and the cortex. Furthermore, we found differential decreased ATP levels across each brain region in Alzheimer’s disease mouse models. The decreased brain ATP levels may potentially be a result of mitochondrial downregulation of ATP synthase in Alzheimer’s disease.

## 2 Results and Discussion

### 2.1 Exosome Preparation and Encapsulation of DNA Aptamer Sensors into the Exosomes

**Figure 1:**
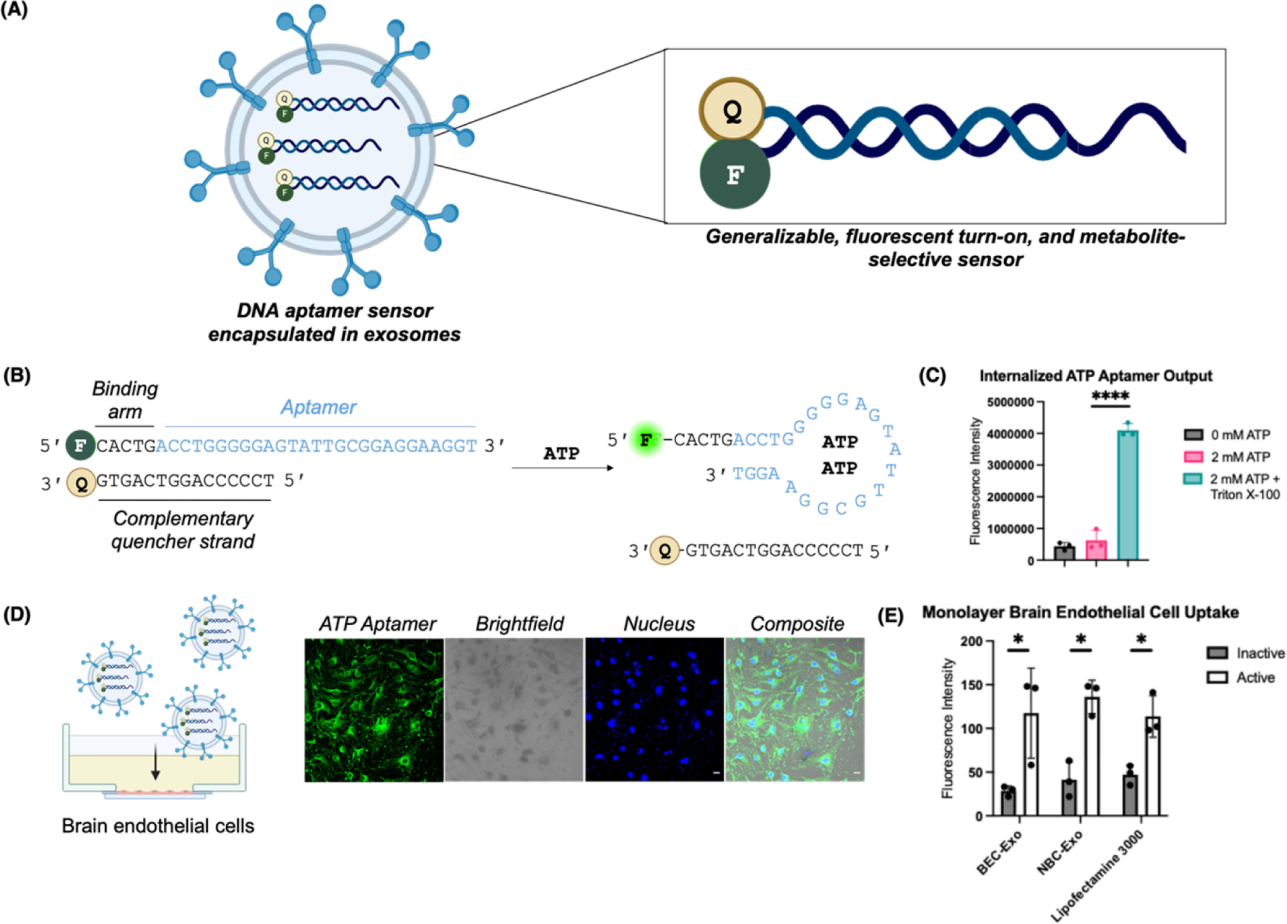
General scheme for encapsulated sensors and monocultured brain endothelial cell delivery studies. (A) Brain cell-derived exosomes innately express surface markers for BBB recognition and cell uptake. FNA sensors can be internalized within these exosomes for targeted BBB crossing and live brain tissue delivery. (B) For aptamers, target metabolite binding results in a structure switch, strand dehybridization, and fluorescence signal turn on. A representative example is the ATP aptamer, in which the fluorophore is marked as F and the quencher is marked as Q. The known ATP aptamer sequence is noted in blue, while the binding arm extension and complementary quencher strand are noted in black. (C) Fluorescence spectroscopy results of the ATP aptamer sensor encapsulated into NBC-Exos in the presence of 0 mM ATP, 2 mM ATP, and 2 mM ATP plus 0.1% Triton X-100 to disrupt the exosomal lipid membrane. (D) Representative confocal microscopy images of NBC-Exos delivering the ATP aptamer sensor into monolayer brain endothelial cells that have been stained with the nucleus-localizing Hoechst 33342 (blue fluorescence). (E) Quantified intensity for the inactive control and ATP aptamer sensor delivered into bEnd.3 cells with BEC-Exos, NBC-Exos, and Lipofectamine 3000. Scale bars: 100 nm. Data shown are *n* = 3, with the error as the standard deviation of the mean; *p < 0.05 and ****p < 0.0001 as determined through an independent *t* test.

To achieve in vivo delivery of DNA aptamers through the BBB, we aimed to encapsulate the sensors into exosomes to allow for targeted delivery and sensor release (Figure 1A). We first isolated native exosomes from two mammalian brain cells, brain endothelial cells (referred to as BEC-Exo) and brain neuroblastoma cells (referred to as NBC-Exo). To determine the successful exosome isolation, we analyzed the size and purity of the cells through Nanosight. We found that both BEC-Exo and NBC-Exo had similar diameters of 110 nm, typical of successful exosome isolation (Figure S1). To demonstrate the effectiveness of such a delivery system, we chose an aptamer for ATP, as ATP is an essential energy-related molecule in the brain.^13,17^ The ATP aptamer sensor consists of an aptamer strand (modified with a fluorophore at the 5′ end) hybridized with a complementary strand (modified with a quencher at the corresponding 3′ end to the aptamer’s fluorophore) (Figure 1B). The aptamer (blue text) is extended by a short binding arm (black text) to allow the complementary strand (black text) to block the aptamer’s binding pocket while ensuring a high melting temperature to prevent background dehybridization at 37 °C (Figure 1B). In the absence of ATP, the quencher’s close proximity to the fluorophore results in minimal fluorescence. ATP binding outcompetes the complementary strand’s hybridization, resulting in the dehybridization of the complementary strand from the aptamer sensor and increased fluorescence. To eliminate artifacts of fluorescent signal fluctuations in the absence of ATP, such as sensor degradation, we designed an inactive control by randomly scrambling the sequence of the known binding motif of the ATP aptamer^17^ to eliminate ATP binding. We ensured that the predicted melting temperature between this inactive control and its complementary quencher strand was similar to that of the hybridized active ATP aptamer sensor. We used pH-insensitive fluorophores (Rhodamine Green for cellular work and Cy5 for in vivo studies) to avoid fluorescent signal fluctuations due to intracellular pH variations such as lysosomal sequestration. As a result of the unique fluorophores, we used their corresponding quenchers: Black Hole Quencher-1 for Rhodamine Green and Black Hole Quencher-3 for Cy5. To minimize background fluorescence, we titrated the respective complementary quencher strands to the active sensor. We found that 1.2 equiv of quencher to aptamer resulted in maximum background suppression with the least amount of quencher strand equivalents. In vitro fluorescence assays with the hybridized strands showed that the optimal active ATP aptamer sensor displayed a linear fluorescence increase in response to an intracellular range of 0–5 mM ATP, with a limit of detection of 0.06 mM ATP (Figure S2). Moreover, the inactive control failed to exhibit a fluorescence increase over 0–5 mM ATP, indicating the active sensor was an accurate measure of the ATP levels (Figure S2).

To load the aptamer sensor inside the exosomes with the highest efficiency, we evaluated several techniques, including incubation, sonication, electroporation, and freeze–thaw.^34−36^ To monitor the exosome loading efficiency, we used the ATP aptamer modified with 5′ Rhodamine Green without a hybridized quencher strand. To separate the internalized aptamer from the excess unloaded aptamer, we performed successive ultrafiltration using a 50 kDa Amicon filter due to differences in the molecular weights of the loaded exosomes compared to the free aptamer. In order to quantify the loading efficiency, we measured the fluorescence intensity of the fluorophore-labeled aptamer in the exosomes versus the total amount of aptamer at the beginning of the protocol. We found that the freeze–thaw method has the highest loading efficiency of 45% (Figure S2), likely because this method resulted in exosome membrane recombination that may favor the internalization of large DNA molecules, while electroporation and sonication only generated transient pores in the exosome membrane.^29,34^ We compared the exosome size before and after the loading process using Nanosight and found minimal changes, indicating that the loading process had low perturbation to the native exosome structure (Figure S3).

To test if the aptamer sensor could still respond to its targets after exosome encapsulation, we used the above optimized loading procedure to load the ATP aptamer sensor (Figure 1B) into the exosomes. Without the addition of ATP, the sensor encapsulated in the exosomes displayed minimal fluorescence signal (Figure 1C) due the quencher’s proximity to the fluorophore. Upon adding 2 mM ATP, we still observed a minimal fluorescence change (Figure 1C). We hypothesized that the lack of the sensor response was due to the successful internalization of the ATP aptamer sensor within the exosomes, as ATP cannot passively cross lipid membranes.^37^ To improve ATP’s penetration into the exosome, we added 0.1% Triton X-100 to deplete the exosome lipid membrane and expose the ATP aptamer sensor. After incubating the lysed exosome solution with 2 mM ATP, we observed a ∼4-fold increase in fluorescence intensity (Figure 1C). This result indicated that the aptamer sensor had been successfully encapsulated into the exosomes and was capable of sensing physiologically relevant intracellular ATP levels after being released into the ATP-containing solution. While the fluorescence assay verified both sensors were still intact after loading, we wondered if there was partial degradation of the sensor during the loading process. After performing each of the four different loading techniques on the ATP aptamer, we analyzed the aptamer on a denaturing gel and observed no detectable fragmentation or loss of the intact aptamer (Figure S3). Together, these results suggest that by encapsulating the aptamer sensors within the exosomes, we can protect the sensor from nuclease degradation and prevent premature sensor activation during the delivery process.

### 2.2 Delivery of the Sensor-Loaded Exosomes to Monocultured Brain Endothelial Cells

To test if the exosomes developed in this work can deliver DNA aptamer sensors into brain cells, we incubated the ATP aptamer sensor encapsulated in either BEC-Exos or NBC-Exos with bEnd.3 cells, a commonly used brain endothelial model cell line. bEnd.3 cells are an ideal BBB model cell line, as they are essential in limiting the transport of molecules across the BBB by maintaining high cargo selectivity and tight junctions.^38^ Notably, the aptamer used can bind ATP, ADP, AMP, and adenosine. As ATP is at least 1 order of magnitude higher in intracellular abundance compared to the latter targets, we refer to the sensor’s output as indicative of ATP levels, but signaling may be in response to the lower concentrations of ADP, AMP, and adenosine as well. After a 4 h period of incubation, we observed the green fluorescence signal from the ATP aptamer sensor in the cytosol, as well as on the cell membrane, as demonstrated by the brightfield channel (Figure 1D). We observed no overlap of the sensor’s green fluorescence signal with the blue fluorescence signal from nucleus-staining Hoechst 33342 (Figure 1D), indicating no sensor delivery to the nucleus. These cellular-localizing signals were consistent regardless of whether BEC-Exos or NBC-Exos was used. More importantly, the fluorescence signals observed for the ATP aptamer sensor encapsulated in BEC-Exos or NBC-Exos were >3-fold more intense than that of the inactive aptamer control, which accounted for any signal from sensor degradation and background fluorescence (Figure S4). Compared with a positive control of Lipofectamine 3000, both exosomes displayed similar delivery efficiencies (Figure 1E, Figure S4). These results indicated that both BEC-Exos and NBC-Exos were effective in delivering the ATP aptamer sensor into monocultured brain endothelial cells, the ATP aptamer sensor was still active after the cellular uptake, and the exosome-delivered sensor responded to physiological levels of intracellular ATP.

### 2.3 Exosome-Mediated Delivery of DNA Aptamer Sensors Across an In Vitro BBB Transwell Model

After demonstrating the successful delivery of the sensor-loaded exosomes to monocultured brain endothelial cells, we evaluated their efficacies in delivering the ATP aptamer sensor across an in vitro Transwell BBB model. The Transwell model is a standard static BBB model consisting of brain endothelial cells cultured on an extracellular matrix-coated insert that is suspended in a solution containing neuroblastoma brain cells.^39^ When the brain endothelial cells are grown to 100% confluency on top of the extracellular matrix-coated insert, they simulate the tight junctions of the BBB, serving as a well-validated and widely used model of the BBB.^40,41^ To fully form the tight junctions, we grew the brain endothelial cells on top of the extracellular matrix-coated insert over a 10 day period and then verified tight junction formation using Lucifer Yellow, a dye commonly used to check the integrity of the BBB model system. If the Lucifer Yellow crosses the BBB model system through paracellular diffusion, then it would indicate a lack of tight junction formation that may contribute to false positive crossing of the BBB model.^42^ After incubating Lucifer Yellow in the Transwell brain endothelial cell layer for 5 h, we observed minimal fluorescence signal from Lucifer Yellow in the target neuroblastoma cell layer (Figure S5), thus verifying tight junction formation.

**Figure 2:**
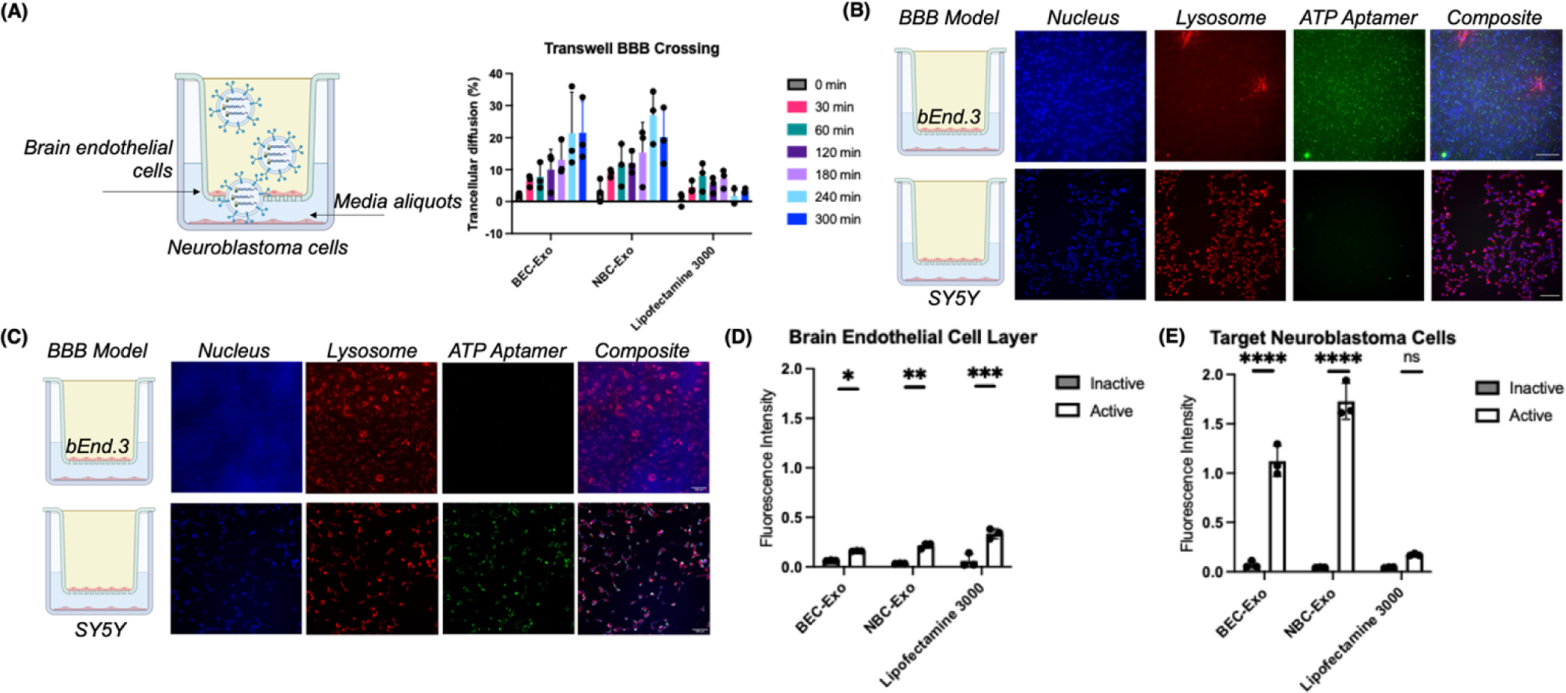
Confocal microscopy and transportation efficiency for Transwell studies with the ATP aptamer. (A) Transportation efficiency of the ATP aptamer sensor measured with media aliquots from the Transwell model over a 5 h period. (B) Live cellular images of the BBB cell model layers with staining for the nuclei (blue fluorescence), lysosomes (red fluorescence), ATP aptamer sensor (green fluorescence), and composite after 2 h of incubation. (C) Live cell images of the BBB cell model layers after exosome-mediated delivery across the BBB model, with staining for the nucleus (blue fluorescence), lysosomes (red fluorescence), ATP aptamer sensor signal (green fluorescence), and composite after 5 h of incubation. (D) Quantified signal of the control and the ATP aptamer sensor with different delivery groups in the BBB cell model layer after 5 h of incubation. (E) Quantified signal of the control and the ATP aptamer sensor with different delivery groups internalized in the target brain cell layer across the Transwell model after 5 h of incubation. Scale bars are 100 nm. Data shown are *n* = 3, with the error as the standard deviation of the mean; *p < 0.05, **p < 0.01, ***p < 0.001, and ****p < 0.0001 as determined through a one-way ANOVA between control and active sensors.

To quantitatively evaluate transportation efficiency across the Transwell BBB model, we added the ATP aptamer sensor delivered through Lipofectamine 3000, BEC-Exos, and NBC-Exos to the brain endothelial cell layer and removed small media aliquots from the basolateral neuroblastoma cell media over multiple time points. We then measured the Rhodamine Green fluorescence signal at each time point to determine the BBB crossing ability. For the first 4 h, all samples displayed increasing fluorescence intensities in the basolateral well media. The permeability of both BEC-Exos and NBC-Exos peaked at ∼30% efficiency at the 4 h time point (Figure 2A). After 4 h, the aptamer fluorescence signal decreased due to the sensor’s uptake into the target neuroblastoma cells. Compared to the Lipofectamine 3000 control, both BEC-Exos and NBC-Exos had nearly 4-fold higher transportation efficiency (Figure 2A).

After demonstrating the high transportation efficiency by measuring the fluorescence signal of the media aliquots from the basolateral neuroblastoma cell media, we used confocal microscopy to visualize the sensor delivery across both the brain endothelial cell layer and the neuroblastoma cell layer. After 2 h of incubation, while we observed a green fluorescence signal from the ATP aptamer sensor in both cell layers of the BBB Transwell model, the majority of the sensor’s signal was still internalized in the brain endothelial cells (Figure 2B). At the 5 h time point, the sensor fluorescence in the brain endothelial cell layer decreased, with a simultaneous increase in the sensor fluorescence in the neuroblastoma layer (Figure 2C). Ultimately, we observed up to a 3.5-fold enrichment of the fluorescence signal in the neuroblastoma cells compared to the brain endothelial cells (Figure 2D and E, Figure S6). After this delivery process, the ATP aptamer sensor displayed a significant signal increase compared to the inactive control, indicating the sensor could still respond to physiological ATP levels after BBB crossing with minimal degradation. This observation was consistent with our monocultured cell data (Figure 1E, Figure S4) and suggested a targeted sensor delivery mechanism to penetrate the BBB. In contrast, when using Lipofectamine 3000 as a delivery agent, the fluorescent signal from the ATP aptamer sensor remained in the brain endothelial cell layer (Figure 2D), and only a small fraction of the aptamer’s fluorescent signal was observed in the target neuroblastoma cell layer (Figure 2E). Therefore, even though Lipofectamine 3000 was equally effective as BEC-Exos and NBC-Exos in sensor delivery to the monolayer brain endothelial cells, the BBB model’s tight cargo selectivity requirements prevented nontargeting Lipofectamine 3000 from delivering the sensor across the BBB model. This supported the hypothesis that brain cell-derived exosomes could deliver DNA aptamer sensors due to their innate surface modifications. After 8 h of incubation, the fluorescence intensities for all samples dropped significantly in both cell layers, indicating sensor degradation (Figure S7). In addition, MTT assays of both BEC-Exos and NBC-Exos showed no significant cell death for either the brain endothelial or neuroblastoma cell lines (Figure S8), suggesting that the fluorescence signal increases were not an artifact of cellular death. Together, these results indicate that exosomes can deliver the DNA aptamer sensor across the BBB model to image intracellular levels of important metabolites like ATP.

### 2.4 Mechanism for the Exosome BBB Delivery Efficiency

**Figure 3:**
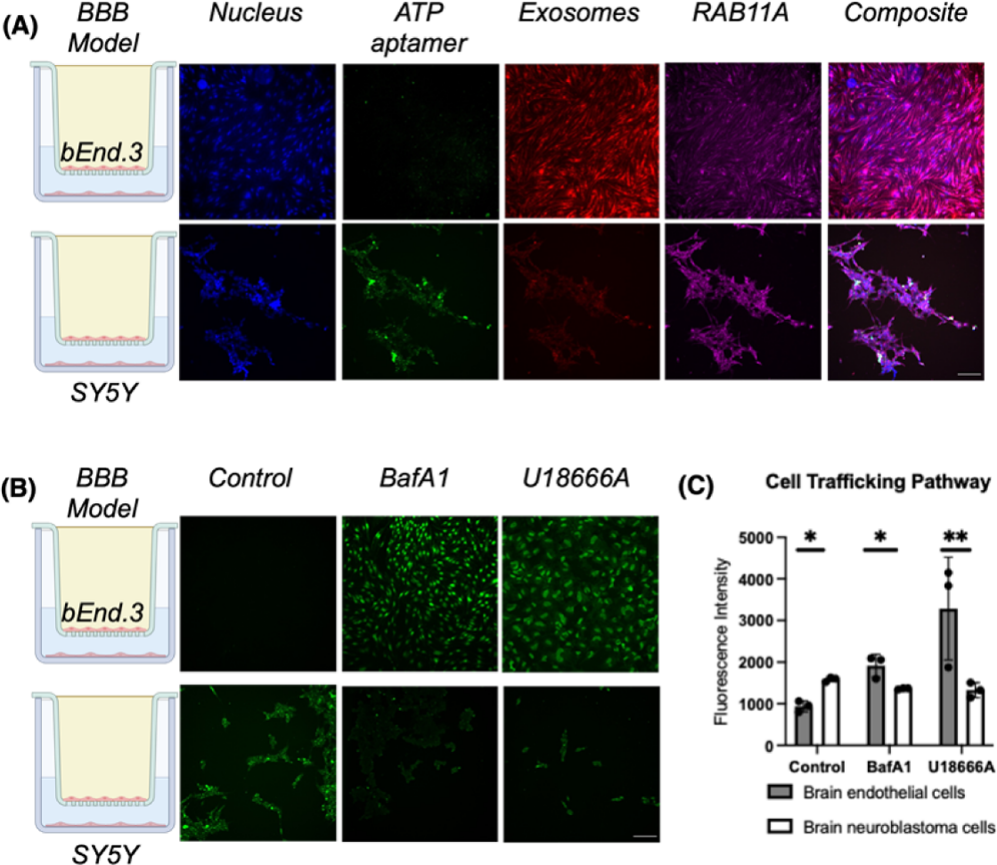
Visualizing the exosomes exploiting the recycling endosome pathway for high ATP aptamer sensor delivery efficiency across the Transwell BBB model. (A) Confocal microscopy images to demonstrate high colocalization of exosomes (red fluorescence) with Rab11a (purple fluorescence) in the bEnd.3 cell layer (top) but not in the SY5Y cell layer (bottom). The blue channel indicated nuclei, and the green channel indicated the ATP aptamer sensor. (B) Modulation of the recycling endosome pathway changed the ATP aptamer sensor signal (green fluorescence) distribution in the Transwell BBB model. (C) Quantified ATP aptamer intensity with the modulation of the recycling endosome pathway in the BBB model. All scale bars are 200 nm. Data shown are *n* = 3, with the error as the standard deviation of the mean; *p < 0.05 and **p < 0.01 as determined through an independent samples *t* test between cell layers.

To explain why the exosomes delivered the sensors with a higher delivery efficiency than Lipofectamine 3000 in the Transwell BBB model, we stained NBC-Exos with lipophilic general membrane PKH26 dye, loaded the stained exosomes with the ATP aptamer sensor, and incubated the system in the BBB model. We chose to study NBC-Exos’ trafficking due to their higher delivery efficiencies than BEC-Exos, as shown in the earlier Transwell BBB experiments (Figure 2E). Surprisingly, over 90% of the stained exosomes’ signal (red fluorescence) persisted in the brain endothelial cells, with limited ATP aptamer sensor signal (green fluorescence) (Figure 3A). Meanwhile, the ATP aptamer sensor signals were internalized in the target neuroblastoma cells, but there were few stained exosomes in these cells (Figure 3A). These results suggested that the exosomes potentially circumvented traditional endolysosomal sequestering of delivered nucleic acids. A possible mechanism for this observation was the recycling endosome pathway, in which cargo to be delivered is repacked inside of intracellular recycling endosomes, which then redirect the cargo back to the plasma membrane, either away or toward the target brain cells.^43,44^

To evaluate the correlations of the sensor-loaded exosomes with the recycling endosome pathway in the Transwell model at the optimal 5 h time point, we visualized the signal colocalization of NBC-Exos and the ATP aptamer sensor with Rab11a, the main protein regulator of endosomal recycling.^43^ We found that the NBC-Exo signal (red fluorescence) was more colocalized with the Rab11a antibody (purple fluorescence) in the brain endothelial cell layer, with a Pearson correlation coefficient of 0.71 ± 0.09 (Figure 3A), than in the target neuroblastoma cell layer (0.48 ± 0.04). In contrast, the Pearson correlation coefficient between the ATP aptamer sensor (green fluorescence) and the Rab11a antibody (purple fluorescence) is higher in the target neuroblastoma cell layer (0.69 ± 0.07 vs 0.48 ± 0.04) than in the brain endothelial cell layer (0.53 ± 0.03 vs 0.71 ± 0.09) (Figure 3A). To further confirm if the exosomes used the recycling endosome pathway, we then modulated the cells with two separate recycling endosome reprogrammers before adding the sensor-loaded exosomes to the BBB model. First, with bafilomycin A, which is a V-ATPase inhibitor that prevents the acidification of endosomes and lysosomes, we observed a nearly 2-fold higher signal from the ATP aptamer sensor in the brain endothelial cells over the control with no pathway modulation (Figure 3B and C, Figure S10). This result indicated the effects of lysosomal sequestration on our delivery system. Second, because recycling endosomes uptake cholesterol and sequester Rab9, the mediator of endosome-Golgi body transport, we used U18666A, an amphiphile to trigger the accumulation of cholesterol in late endosomes, thus hindering multivesicular bodies and the recycling endosome pathway.^45^ With U18666A, we found a 3.5-fold increase in the ATP aptamer sensor signal in the brain endothelial cells, while the aptamer sensor signal in the neuroblastoma cells decreased by 20% (Figure 3B and C). The higher aptamer sensor signal sequestration in the brain endothelial cells when the recycling endosome pathway was modulated suggested that the exosomes favor the recycling endosome pathway. At the 2 h time point, recycling endosome modulation increased the aptamer signal in the brain endothelial cells but had negligible effect on signal in the brain neuroblastoma cells, likely due to incomplete sensor delivery across the BBB model at this early time point (Figure S9).

### 2.5 Delivery of DNA Aptamers Across the BBB In Vivo for Sensing of Brain ATP Levels

**Figure 4:**
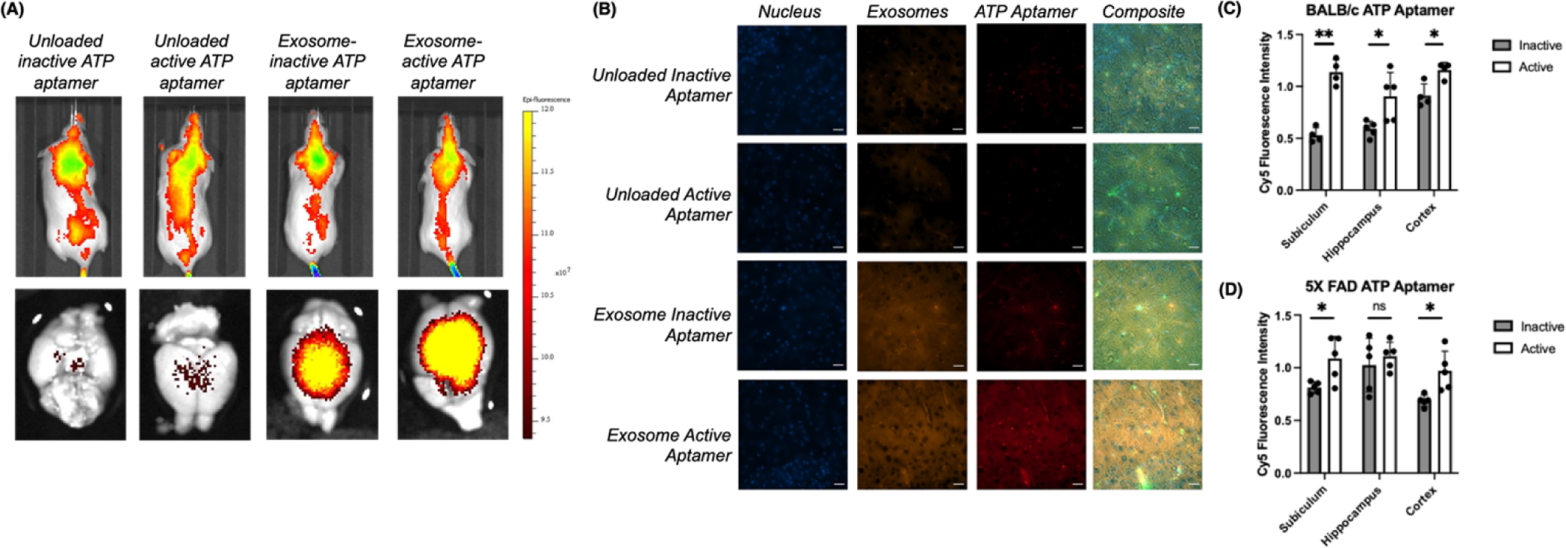
Delivery of the DNA aptamer for ATP cross the BBB of live mice and stained brain slice images after tail vein injection. (A) In vivo imaging of BALB/c mice. Scale is the radiant efficiency [p/sec/cm/sr]/[μW/cm^2^]. (B) Representative images of NBC-Exos internalized in the cortex of the deep brain tissue with the ATP aptamer sensor output in BALB/c mice. The blue channel indicated nuclei, the orange channel indicated stained exosomes, the red channel indicated the aptamer, and the composite was the merged frame. Scale bars: 20 μm. (C) Quantified ATP aptamer sensor intensities in different brain regions for both BALB/c and 5XFAD mice. Data shown were collected with three biological replicates per sensor and, for both BALB/c and 5XFAD mice, with 3 images taken per brain region, with the error as the standard deviation of the mean; *p < 0.05 and **p < 0.01 as determined through an independent samples *t* test between control and active sensors.

While the Transwell model is a conventional and widely accepted model of the BBB for in vitro studies, static models may not accurately mimic the BBB’s tightly regulated nature in vivo. With the preliminary evidence that the exosomes could deliver the active DNA aptamer sensor across the BBB Transwell model with high efficiency, we subsequently examined the exosome system’s delivery in live mice. Because the NBC-Exos resulted in the highest delivery efficiency across the Transwell BBB model, we used them for in vivo studies as well. After loading the NBC-Exos with the Cy5-labeled ATP aptamer sensor, we performed tail-vein injections of the exosome solution into healthy BALB/c mice. To account for potentially false positive signals due to sensor degradation, we used an inactive control containing a scrambled sequence modified with a Cy5 label. To account for different delivery efficiencies to different cell types, we labeled the exosomes with a general membrane PKH26 dye to track the exosomes simultaneously with the sensor. To obtain a delivery timeline, we monitored whole-body fluorescence through the Xenogen IVIS system over the course of 48 h for all samples. As negative controls, we also performed tail vein injections of the sequences without exosome encapsulation (unloaded inactive and unloaded active ATP aptamer, respectively, hereafter). With these unloaded controls, the fluorescence signals remained mainly in the circulation system without brain region accumulation (Figure 4A). In contrast, for both the inactive sequence and active ATP aptamer encapsulated in the NBC-Exos, we observed signal accumulation in the brain region at 6 h (Figure 4A). Periodic imaging over the next 48 h showed a general reduction in fluorescence levels from the whole-body circulation for all groups (Figure S11). Because the whole-body imaging provided promising results at 6 h postinjection but IVIS only imaged up to 20 μm in depth, we excised the live brain tissue at this time point. We imaged the live brains on the IVIS and found 1.5-fold greater fluorescence intensity for the exosome-mediated samples compared to the unloaded controls, which accounted for background fluorescence in the blood vessels (Figure 4A). Likewise, the ATP aptamer sensor displayed a higher Cy5 fluorescence intensity than the inactive control for the NBC-Exo-mediated groups (Figure 4A). Biodistribution assays of the major organ systems found that the exosome samples displayed higher brain accumulation compared to traditional synthetic lipid nanoparticles and unloaded sensor controls, indicating successful brain internalization (Figure S13). As living systems face major pharmacokinetic obstacles such as liver and kidney accumulation of unwanted agents, serum protein interference, short half-life of injected drugs in the bloodstream, and the multitude of cell types involved in regulating the BBB, we expected that the signal output in mice would be lower than that predicted by the in vitro BBB model.^21,22^

### 2.6 Imaging ATP Distributions in Brain Slices of Healthy and Alzheimer’s Disease Model Mice

To evaluate ATP levels and cellular locations with higher spatial resolution, we sliced and imaged fresh frozen whole brains after BBB delivery. We visualized the stained exosomes (orange fluorescence) and sensors (red fluorescence) distributed throughout the whole brain tissue, including all major brain regions, such as the subiculum, cortex, and hippocampus (Figure 4B, Figures S14–S16). With the ATP aptamer sensor, we found ATP distributed throughout these major brain regions through increased Cy5 signals compared with the inactive control (Figures S14–16). Consistent with the in vitro BBB model (Figure 2), the ATP aptamer sensor did not display signal output in the nuclei (blue fluorescence channel) (Figure 4B). Due to different sensor delivery efficiencies in different brain regions, we normalized the sensor signal to the stained exosome signal for sensor quantification. For the ATP aptamer sensor, we observed the unique accumulation of a 2.1-fold fluorescence signal in the subiculum compared to the inactive control (Figure 4C, Figure S14). The hippocampus and cortex regions also displayed 1.3-fold and 1.5-fold increased fluorescence levels, respectively (Figure 4C, Figures S15 and S16). These results are consistent with imaging mass spectrometry reports that found significantly varied ATP levels throughout different brain regions in fixed tissue.^46^ Of these regions, the subiculum is of special importance, as it interacts with a wide range of cortical and subcortical areas, influencing whole brain activity by integrating hippocampal and cortex brain activity.^47^ Glucose uptake experiments in rats previously found that the subiculum had the highest glucose uptake rates, indicating the subiculum’s high metabolic requirements. Our findings of high ATP in the subiculum were likely linked to the region’s enhanced glycolytic activity.

To further understand the distributions of ATP in brain disorders, we applied the stained NBC-Exos to deliver the sensors in Alzheimer’s disease 5xFAD mouse models. Likewise, the ATP aptamer sensor displayed significant fluorescence signal turn-on throughout the subiculum, hippocampus, and cortex (Figures S12 and 17–19). To account for variable delivery efficiencies in different brain regions, we normalized each sensor to the stained exosome signal for quantification. While the subiculum and the cortex displayed statistically significant ATP aptamer sensor output increases compared to their inactive controls of 1.3-fold for the subiculum and 1.4-fold for the cortex, the fold changes were lower compared to those of the same sensors applied in healthy mice, indicating lower levels of ATP (Figure 4D). Using a positive control of ATP-dependent luciferase to assess whole-brain ATP levels, we found that the amount of ATP in the 5X-FAD mouse brain decreased compared to that of the healthy mice, confirming the aptamer’s robustness (Figure S20). Other studies have also found reduced ATP synthesis in Alzheimer’s disease cell models.^48^ Potentially, the lower levels of these energy-associated metabolites may be due to dampened mitochondrial oxidative phosphorylation, a biomarker of Alzheimer’s disease.^49,50^ Degrees of mitochondrial oxidative phosphorylation may vary depending on the cell type and brain region, highlighting the importance of spatial imaging in intact live tissue, which is not accomplished by many state-of-the-art methods.

## 3 Conclusions

To image energy-related metabolites in live brain tissue, we developed a generalizable method to deliver a DNA aptamer sensor to cross the BBB of live mice. Using brain cell-derived exosomes, we encapsulated an ATP-responsive aptamer sensor to allow for targeted delivery across the BBB and sensor release in the brains of live mice. Because the exosomes are derived from brain cells, they outperformed state-of-the-art nucleic acid delivery systems, as demonstrated in the classic BBB Transwell model and in live mice. We found evidence that the system uses recycling endosomes to transfer the sensors between the delivered exosomes and native endosomes, resulting in its high delivery efficiency. The successful BBB crossing and sensor delivery allowed us to sense physiologically relevant levels of ATP. After sensor output normalization with respect to exosome delivery efficiency, we quantified relative amounts in different brain regions such as the subiculum, hippocampus, and cortex. To evaluate how these targets change with diseases, we applied our system with 5xFAD Alzheimer’s mouse models and found that ATP levels decrease with neurodegeneration. While previous Alzheimer’s research studies have also found decreased levels of both targets in whole brain tissue and patient serum, we were able to provide spatial resolution in live tissue, demonstrating that the decreased levels of ATP were not uniform throughout the brain. These results demonstrated the importance of the brain region type to contributing to disease pathology. A limitation of this work is the aptamer’s binding to other adenosine-containing molecules, such as AMP and ADP. Due to the high relative abundance of ATP over AMP, ADP, and adenosine,^51−53^ the majority of the sensor’s response can be attributed to ATP. However, the low concentrations of ADP, AMP, and adenosine also contribute to the aptamer’s signal. Compared to other methods that use fixed tissue samples that do not preserve small molecule metabolite distribution, we performed live tissue delivery and sensing in which the dynamic metabolite flux, tissue architecture, and cell heterogeneity were intact. Our method can be expanded to a wide variety of other aptamer sensors developed for other metabolites.

## Supporting Information Available

Protocols for materials and detailed methods, exosome generation, in vitro sensor and control fluorescence activity, exosome integrity after sensor loading, all raw images from cell studies (monocultured, Transwell BBB, and mouse brain tissue), the Transwell permeability assay, live mice images, additional isolated brain images, biodistribution images, and all DNA sequences used (PDF)
