# Supplementary material for: Delivering DNA Aptamers Across the Blood–Brain Barrier Reveals Heterogeneous Decreased ATP in Different Brain Regions of Alzheimer’s Disease Mouse Models
Source: ACS Cent Sci. 2024 Jul 31;10(8):1585–93. doi: 10.1021/acscentsci.4c00563 (PMC11363336; doi:10.1021/acscentsci.4c00563)
Supplement: Supplementary file 1 — oc4c00563_si_001.pdf [file oc4c00563_si_001.pdf]

Delivering DNA Aptamers Across the Blood-Brain Barrier Reveals Heterogeneous Decreased ATP in Different Brain Regions of Alzheimer's Disease Mice Models

Mandira Banik[1], Aaron P. Ledray[1], Yuting Wu[1], and Yi Lu[1*]

1. University of Texas at Austin, Department of Chemistry, Austin, TX 78712, United States

* Corresponding author. Email: yi.lu@utexas.edu

Table of Contents

Methods **2**

DNA Sequences **8**

Supporting Figures **9**

Methods

General materials and instruments

All oligonucleotides were purchased from Integrated DNA Technologies. bEnd.3 (CRL-2299) cell lines were purchased from American Type Culture Collection, while SH-SY5Y were a gift from Dr. Liviu Mirica's lab at the University of Illinois at Urbana-Champaign. Cells were screened for pathogens by IDEXX BioAnalytics using IMPACT 3, with no pathogens found. Female healthy BALB/cJ (RRID:IMSR_JAX:000651) were purchased from the Jackson Laboratory. Female Alzheimer's disease mice models B6.Cg-Tg(APPSwFlLon, PSEN1*M146L*L286V) 6799Vas/Mmjax, RRID:MMRRC_034848-JAX, were purchased from the Mutant Mouse Resource and Research Center (MMRRC) at The Jackson Laboratory, an NIH-funded strain repository, and was donated to the MMRRC by Robert Vassar, Ph.D., Northwestern University. All mice were housed under standard SPF level housing (12 hours light, 12 hours day, fed ad libitum) at UT Austin's animal resource facility, with all protocols approved by UT Austin's Institutional Animal Care and Use Committee. MilliQ water was used for all buffer preparation. Reagents and chemicals were purchased from Sigma-Aldrich or Fisher Scientific at the highest purity available. Antibodies were purchased from Invitrogen. Fluorimetry experiments were performed with a Horiba fluorimeter, confocal microscopy images were collected on either Zeiss 710 laser scanning microscope (monocultured cells) or Nikon spinning disk microscope (Transwell and brain tissue), and live mice images were collected with the Xenogen IVIS instrument. No unexpected safety hazards were encountered.

Cell Culture

bEnd.3 cells were cultured in DMEM (Corning) with 10% fetal bovine serum (Corning) with 100 U/mL penicillin-streptomycin (Gibco). SH-SY5Y cells were cultured in 50/50 EMEM and Ham-F12 (Corning) with 10% fetal bovine serum and 100 U/mL penicillin-streptomycin. All cells were incubated at 37°C in 5% CO_2. Once passage 30 was reached, a new cell stock was thawed.

Preparation and characterization of cell-derived native exosomes

To generate exosomes, the bEnd.3 or SH-SY5Y cells were plated at 90% confluency in T75 flasks. After one day, the standard cell culture media was replaced with conditioned cell culture media predepleted of exosomes. Conditioned cell culture media was generated by purified out bovine exosomes using the Total Exosome Isolation Kit (Sigma Aldrich). Briefly, the cell culture media was centrifuged at 2000*g* for 5 minutes at room temperature. 5 mL of the precipitation reagent was added to the 10 mL of the cell culture supernatant in a 15 mL conical tube. The mixture was incubated overnight at 4°C and then spun at 10,000*g* for 1 hour at 4°C. The supernatant depleted of exosomes was used as the media for exosome generation. After two days, the cell media was transferred to a 15 mL conical tube. Exosomes were isolated with Sigma-Aldrich Total Exosome Isolation kit. Briefly, after the two day period, the cell culture media was centrifuged at 2000*g* for 5 minutes at room temperature to remove cell debris. 5 mL of the precipitation reagent was added to the 10 mL of the cell culture supernatant in a new 15 mL conical tube. The mixture was incubated overnight at 4°C and then spun at 10,000*g* for 1 hour at 4°C. The resulting white precipitate was resuspended in 1 mL sterile PBS and syringed multiple times to remove clumps.

Verification of exosome isolation through Nanosight

Exosome size, purity, and concentration were characterized by Nanosight NS500 (Center for Electrochemistry, UT Austin). 5 µL of the resuspended exosome stock was diluted in 1000 µL

PBS buffer. Run times of 1 minute, with the camera level set to 8 at room temperature and screen gain set to 1, were used to collect size and purity data. Data tracking and analysis was automatically performed by the Nanoparticle Tracking Analysis software on the Nanosight. The Center for Electrochemistry used calibration beads (Malvern) to calibrate the instrument before each run.

Sensor hybridization

For the ATP aptamer sensor, 10 µM stocks of annealed sensor was prepared by mixing the aptamer to quencher strand at a 1:1.1 ratio in PBS + 2 mM $MgCl_2$. The strands were annealed following a thermocycler method of heating at 90°C for 5 minutes and then cooled to 25°C over 40 cycles. Unless otherwise noted, all cellular and in vivo experiments used the hybridized sensors, noted as the ATP aptamer sensor. Control strands (scrambled sequence for the ATP aptamer) were hybridized with the same protocol). For these controls, all cellular and in vivo experiments used the hybridized strands and were labeled as inactive control.

Loading efficiency measurements of exosomes with the ATP aptamer

Multiple methods for internalizing the sensor within the exosomes were attempted. For all methods, 1.25 µM ATP aptamer modified with a 5′ Rhodamine Green fluorophore was incubated in a 250 µL solution of 1 X 10^6 exosomes (particle count based upon Nanosight measurements) at room temperature for 30 minutes. Any excess volume required was made up with PBS + 2 mM $MgCl_2$. For the incubation loading technique, no further steps were performed. For freeze-thaw, the samples were then flash frozen in liquid nitrogen and allowed to warm in a heated water bath. The freeze-thaw process was performed for a total of 6 cycles. For sonication, the samples were sonicated for 6 cycles at 100% power, 37 kHz of 20 second sonication, with 5 second rest periods between each sonication pulse. For electroporation, the samples were electroporated at 200 mV for 6 cycles, with 5 second rest periods between each pulse. For all methods, the unloaded aptamer was then removed from loaded exosomes with 50 kDa MWCO Amicon ultrafiltration tubes by 3X centrifugation at 4500*g* for 10 minutes. The removal of unloaded sensor was visually evaluated by the presence of a light yellow solution in the flow-through. Fluorescence signal post loading was determined by incubating the loaded exosomes with 0.1% Triton-100 for 10 minutes before fluorescence emission analysis by fluorimetry (Horiba Fluoromax, excitation: 480 nm, emission: 520 nm, slit widths 5 mm). To generate a standard curve, we prepared a dilution range of 200 µL solutions of 50 nM to 1 µM Rhodamine Green-labeled ATP aptamer in 50 mM Tris, 100 mM NaCl, 5 mM $MgCl_2$ pH 7.4 at 37°C. We measured these fluorescence emissions by a Horiba Fluoromax (excitation: 480, emission: 520, slit widths 5 mm) to generate a standard curve of fluorescence intensity versus known Rhodamine Green-labeled ATP aptamer concentration. To determine the loading efficiencies, we used the standard curve's linear best fit equation with fluorescence signal from the loaded exosome to calculate the concentration of aptamer in each exosome solution.

Cell viability assay

The cytotoxicity of the ATP aptamer sensor loaded exosome systems was evaluated by the standard MTT assay. Cells (bEnd.3 and SY5Y) were grown until 90% confluency in 96 well plates. Stock solutions of exosomes were prepared in cell culture grade water, with gradient dilutions to prepare different concentrations of native and surface modified exosomes from both SY5Y cells and bEnd.3 cells. On the day of the experiment, media was replaced with fresh Opti-MEM. A negative control of only Opti-MEM was used. A positive control was 200 µL of fresh 30%

hydrogen peroxide. The serial dilution of exosomes was added to each well, in triplicate, and incubated at 37°C for 4 hours in the cell growth chamber. A solution of MTT (stock 5 mg/mL in PBS) was diluted in Opti-MEM to a final concentration at 0.25 mg/mL and 100 μL was added to each well after aspirating exosome containing solutions. The plates were then incubated for 5 hours at 37°C. The MTT solution was then aspirated and replaced with 200 μL DMSO, and the absorbance was measured at 570 nm using a BioTek plate reader.

Monocultured cell delivery assays

Cells (bEnd.3 or SY5Y) were plated in ibidi 8 well plates (for imaging). Exosome solutions were normalized based on BCA assays, in which the total probe-loaded exosome solution was ~5.7 μg total protein per 0.5 mL cell culture media, corresponding to ~200 nM functional-DNA loaded. As a comparison to commercially available transfection agents, the manufacturer's instructions were followed for liposome complex formation with Lipofectamine 3000 and Turbofect and 400 nM DNA. Briefly, for Lipofectamine 3000, two separate tubes were prepared: 1, 25 μL Opti-MEM with 1 μL Lipofectamine 3000 (vortex 3 seconds) and 2, 25 μL Opti-MEM + 200 nM DNA). Tubes 1 and 2 were mixed and incubated at room temperature for 3 minutes. Then 50 μL Opti-MEM was added and the solution was incubated for 12 minutes at room temperature before being added to the cell culture media. For Turbofect, 50 μL of Opti-MEM were mixed with 1 μL Turbofect and 200 nM DNA at room temperature for 15 minutes before being added to the cell culture media. Exosome solutions were allowed to incubate with the cells for 4 hours in the humidified cell culture incubator at 37°C. Before imaging, cells were washed 3X with PBS. 200 μL fresh DMEM without phenol red was added to each well. Lasers of 405 nm (collect 410 nm to 480 nm), 488 nm (collect 490 to 550 nm), and 561 nm (collect 570 to 630 nm) were used. The pinhole was set to 1 Airy unit. All microscope settings were kept constant. All images shown use the same minimum and maximum settings.

Exosome mediated delivery in the Transwell BBB model

Transwell membrane inserts (24 mm, 0.4 μm pores, polyester TC-treated, Corning) were prewarmed with 1 mL bEnd.3 cell culture media (DMEM), in which the apical side was prewarmed with 2 mL SY5Y cell culture media (EMEM/Ham's F12). bEnd.3 cells (0.3 X 10^6) were seeded in the apical side of the insert and SY5Y (0.3 X 10^6) were seeded in the basolateral side. The cells were cultured at 37°C in 5% CO_2 for 10 days, or until 100% confluency for the bEnd.3 cell layer. On the day of the delivery experiment, the media was replaced with fresh Opti-MEM and Rhodamine Green labeled ATP aptamer sensor loaded exosome solution (5.7 μg total protein per 0.5 mL cell culture media, corresponding to ~200 nM sensor loaded.) was added to each the apical side. Native and surface modified SY5Y and bEnd.3 exosomes were used. For imaging, after 4 hours of incubation in a humidified cell culture incubator at 37°C, 75 nM of Lysotracker Red was added to each well and allowed to incubate for 1 hour. After 1 additional hour of total incubation in a humidified cell culture incubator at 37°C, the cells were washed 3X with PBS and replaced with 1 mL fresh DMEM without phenol red and 1 μg/mL Hoescht 33342. Positive controls include adding the probe loaded exosome solution to the basolateral neuroblastoma cell media directly and Lipofectamine 3000-sensor to the basolateral side directly. For Transwell transportation efficiency assays measured over time, the exosomes were allowed to incubate over 5 hours. Each Transwell BBB integrity was verified with Lucifer Yellow penetration. A stock solution of 60 μM Lucifer Yellow in Hanks Balanced Salt Solution with 1% DMSO was prepared. Lucifer Yellow was added to the bEnd.3 cell layer at a final concentration

of 0.1 mg/mL. At each time point, 150 µL of the cell culture media from the SY5Y layer was transferred to a clear bottom 96 well plate (Corning). The plate was read in a BioTek plate reader with excitation: 430 nm, emission 535 nm. Controls of Hank's Balanced Salt Solution buffer and 0.1 mg/mL Lucifer Yellow were used. The percent permeability was calculated as (sample-blank)/(lucifer yellow-blank) * 100. Lucifer Yellow assays repeatedly showed less than 2% permeabilization across the brain endothelial cells, which is in the acceptable window of endothelial cell leakiness. All imaging studies were performed with live cells, except for the colocalization studies with the recycling endosome pathway. Lasers of 405 nm (collect 410 nm to 480 nm), 488 nm (collect 490 to 550 nm), and 561 nm (collect 570 to 630 nm) were used. The pinhole was set to 1 Airy unit. All microscope settings were kept constant. Images could only be collected with the 10X objective, due to the working distance limitation and large Transwell depth. All images shown use the same minimum and maximum settings.

Recycling endosome pathway modulation

In the Transwell BBB model, both bEnd.3 and SY5Y cells were treated with 0.1 µM Bafilomycin A1 for 30 min in DMEM, 1 µg/mL U18666A for 30 minutes in DMEM, or in DMEM alone for 30 minutes in a cell culture incubator at 37°C. After this initial incubation, sensors were added to the Transwell, as described in exosome-mediated delivery across the Transwell BBB model. Deliveries across the Transwell were performed for 2 hours, 5 hours, and 8 hours in a cell culture incubator at 37°C. Subsequently, the cell fixation for antibody staining along the recycling endosome pathway protocol was followed.

Cell fixation for antibody staining along the recycling endosome pathway

After the exosome mediated delivery in the Transwell BBB model protocol, both the bEnd.3 and SY5Y cells were washed 1X with PBS. Cells were then fixed for 10 minutes with 4% paraformaldehyde and washed 1X with PBS. Both cell layers were permeabilized with 0.2% Triton X-100 for 10 minutes and washed 1X with PBS. Cells were then blocked with 1% BSA in PBS at room temperature for 45 minutes. Cells were then washed 1X with PBS and cells were incubated with 1:100 dilution of Rab11a antibody in PBS (Invitrogen Catalog #71-5300) overnight at 4°C, washed 3X with PBS, and then stained with a 1:1000 dilution of secondary donkey anti-rabbit Alexa Fluor 647 in PBS (Invitrogen Catalog #A-31573) in 1% BSA in PBS blocking solution for 45 minutes. Before imaging, cells were washed 1X with PBS, with fresh PBS added as the imaging solution. Lasers of 405 nm (collect 410 nm to 480 nm), 488 nm (collect 490 to 550 nm), 561 nm (collect 570 to 620 nm), and 633 nm (collect 640 to 660 nm) were used. The pinhole was set to 1 Airy unit. All microscope settings were kept constant. All images shown use the same minimum and maximum settings.

Transportation efficiency study

To study the transportation efficiency of the exosomes across the BBB cell model, we used the Transwell BBB model as described above. At each time point (5 min, 15 min, 30 min, 60 min, 120 min, 180 min, and 240 min), 5 µL of the basolateral side was aliquoted in 195 µL Opti-Mem. The fluorescence signal from the media samples was analyzed with a BioTek plate reader. Opti-Mem was used as the blank, and a negative control of Lipofectamine 3000 + 200 nM Rhodamine Green labeled ATP aptamer sensor added directly to the basolateral side were used. Because the fluorescence intensity is proportional to the functional-DNA sensor, the transportation efficiency of each method was determined as $(F_{time} - F_{medium})/(F_{total} - F_{medium})$, where F_{time} is the signal at

different time points, F_{medium} is the Opti-MEM blank, and F_{total} is the negative control of the DNA directly added to the basolateral side. This was performed 3 times.

Delivery of sensors in live mice

Animal care was approved by and followed the guidelines of the University of Texas at Austin's Institutional Animal Care and Committee (IACUC). For in vivo experiments, 150 µg of exosomes (determined by BCA) were loaded with 25 µmol of Cy5-ATP aptamer sensor/kg of mice body weight (~14 g), with unloaded probe purified from the exosomes with 50 kDa Amicon ultrafiltration tubes the day before the experiment and stored at -20°C overnight. Negative controls of PBS, unloaded inactive sensor, and unloaded active sensor were used. Positive controls of PEG-Liposome In Vivo Transfection Reagent (Altogen Biosystems, were prepared according to manufacturer's protocol) and exosomes with exposed Cy5 ATP aptamer (no hybridized quencher) were used. All samples with lipid carriers (exosomes and liposome control) were stained with PHK26 membrane dye (Sigma-Aldrich) as an internal control for delivery efficiency normalization. 3 month old BALB/c mice (Jackson Laboratory) were used as the healthy control, while 3 month old 5xFAD mice (National Institute of Health, Mutant Mouse Resource and Research Centers, purchased through Jackson Laboratory) were used as the Alzheimer's Disease transgenic model mice. Mice were anesthetized using isoflurane (2%) during injections and in vivo imaging. Tail vein injections were performed with a total volume of 200 µL per mouse using a sterile 22 gauge needle. After injections, mice were allowed to recover in their cage, with access to food and water ad libitum during the experiment timecourse. No adverse side effects for the mice were noted after injections. Mice were sacrificed with CO_2 as the primary method of euthanasia and cervical dislocation as the secondary method of euthanasia.

In vivo imaging

In vivo imaging was performed with a Xenogen IVIS system. Settings used were 640 nm excitation, 680 nm emission, f number 4, binning factor 8, and 6.6 field of view. To first visualize the delivery system's pharmacokinetics, mice were injected with either PBS or with probe loaded exosomes. The isofluorane-sedated mice were imaged over a 48-hour period at 15 minutes, 1 hour, 2 hours, 4 hours, 6 hours, 12 hours, 24 hours, and 48 hours post-injection. Probe signal in the brain region saturated at 6 hours post injection, with a clear decrease in blood circulation over the next two days. All images shown use the same minimum and maximum settings.

Post-mortem tissue analysis for biodistribution

To study the biodistribution of the exosome systems, the main organs of the mice (brain, heart, liver, spleen, lung, and kidney) were excised at 6 hours post-injection. Immediately after excision, the organs were washed with 3X with PBS to remove blood. The organs were imaged using the Xenogen IVIS Spectrum fluorescent imager, with the mean fluorescence signals analyzed using the instrument software. The same settings were used as the in vivo imaging (640 nm excitation, 680 nm emission, f number 4, binning factor 8, and 6.6 field of view).

Post-mortem tissue analysis of brain sections

Six hours post-injection, the mice were sacrificed (primary method: CO_2 and secondary method: cervical dislocation) and the brains excised. The brains were fixed overnight in 4% PFA at 4°C and dehydrated with 30% sucrose in PBS at 4°C for 2 days. After embedding in O.C.T. (Scigen Tissue-PLUS OCT Compound, Fisher Scientific) at -20°C, the brains were sectioned to 30 µm

with a cryostat. Brain slices were stored in cryopreservation media (30% ethylene glycol, 30% sucrose, 0.1 M PBS, pH 7.35). The dissected brain slices were washed in PBS, stained with Hoechst 33342 for 10 minutes, and mounted on SuperFrost Plus slides (Fisher). The tissue samples were covered with a glass cover slip and sealed with clear nail polish prior to imaging. Lasers of 405 nm (collect 410 nm to 480 nm), 488 nm (collect 490 to 550 nm), and 561 nm (collect 570 to 630 nm) were used. All images shown use the same minimum and maximum settings.

ATP luminescent measurements

One three-month old BALB/c female mouse and one three-month old 5x-FAD female mouse were sacrificed using CO_2 as the primary method of euthanasia and cervical dislocation as the secondary method of euthanasia. Their brains were harvested, washed with PBS, and fixed overnight in 4% PFA at 4°C. Brains were then dehydrated with 30% sucrose in PBS at 4°C for 2 days. The whole brain was then suspended in 200 μL PBS in a 1.5 mL Eppendorf tube and flash frozen in liquid nitrogen, allowing the nitrogen to enter the tube. Once frozen, a pestle homogenizer was used to grind the tissue. After complete grinding, the samples were centrifuged at 12,000*g* for 5 minutes to separate the debris. 10 μL of supernatant was added in triplicates to a white opaque 96 well plate (Cayman Chemical) for each sample. Using the Luminescent ATP detection kit (Cayman Chemical), all manufacturer instructions were following for sample preparation and standard curve generation. After sample preparation, samples incubated in the dark at room temperature for 15 minutes before measurement. Luminescence samples were read using a BioTek plate reader, with 1 second integration times.

DNA Sequences

Sequence Name	Sequence (5′ to 3′)
In cells active ATP aptamer	/5RG/CAC TGA CCT GGG GGA GTA TTG CGG AGG AAG GT
In cells active ATP quencher	TCC CCC AGG TCA GTG/BHQ_1/
In vivo active ATP aptamer	/Cy5/CAC TGA CCT GGG GGA GTA TTG CGG AGG AAG GT
In vivo active ATP quencher	TCC CCC AGG TCA GTG/BHQ_3/
In cells scrambled inactive ATP aptamer	/5RG/GTG AAG GTG CAA CAG TCG GCT GGA GTT CGG AG
In cells scrambled inactive ATP quencher	CTG TTG CAC CTT CAC/3BHQ_1/
In vivo scrambled inactive ATP aptamer	/5Cy5/GTG AAG GTG CAA CAG TCG GCT GGA GTT CGG AG
In vivo scrambled ATP quencher	CTG TTG CAC CTT CAC/3BHQ_3/

Table S1: Sequences used in this study. RG indicates rhodamine green fluorophore, BHQ_1 indicates Black Hole Quencher-1, Cy5 indicates cyanine 5 fluorophore, BHQ_3 indicates Black Hole Quencher-3.

Supporting Figures

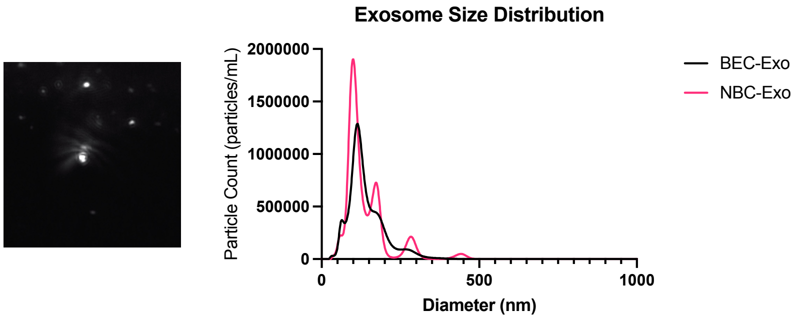

Figure S1: Nanosight to verify exosome size and purity. BEC-Exo and NBC-Exo size, purity, and concentration were characterized by Nanosight NS500. 5 µL of the resuspended exosome stock was diluted in 995 µL of PBS. Run times of 1 minute, with the camera level set to 8 at room temperature and screen gain set to 1 were used to collect size and purity data of the freshly purified exosomes. Both exosome sizes peaked at ~110 nm, with minor exosomal aggregation observe through the doublet and multimer formation.

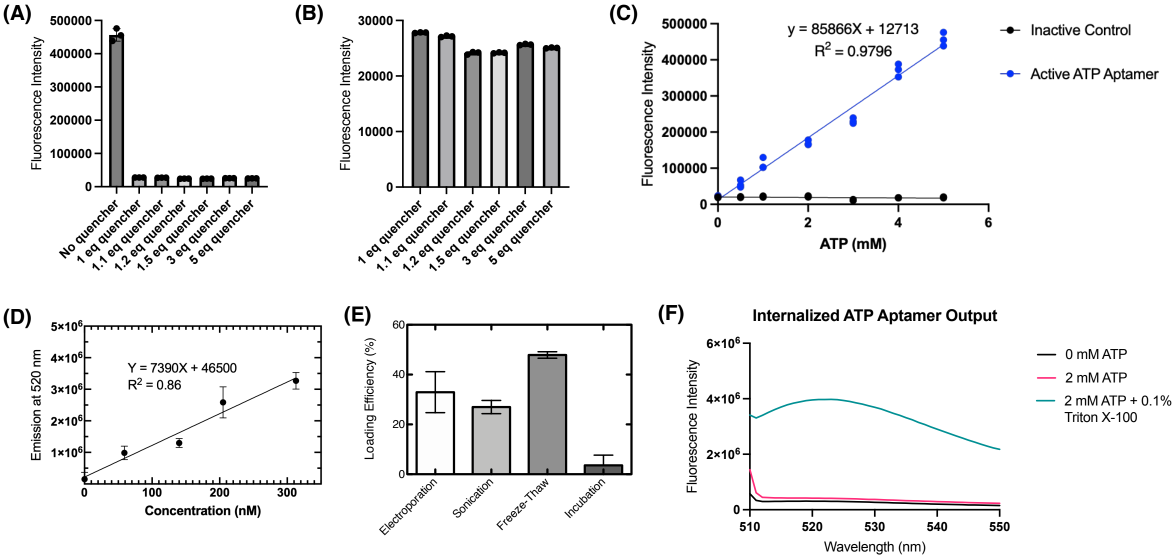

Figure S2: Quantify exosome loading efficiency and exosome encapsulated sensor response. (A) Hybridized ATP aptamer quencher strand optimization compared to a no quencher control. (B) Zoomed in data on Figure S2A to show quenching efficiency optimization. (C) Hybridized ATP aptamer and hybridized inactive control fluorescence intensities compared to ATP concentrations. The limit of detection was calculated by LOD = 3σ/S, where σ is the standard deviation of blank solutions and S is the slope of calibration curve of the active aptamer. This determined a limit of detection of 0.06 mM ATP. (D) Standard curve of fluorescence intensity vs known concentrations of the Rhodamine Green-labeled ATP aptamer. (E) Loading efficiency of different vesicle loading techniques. Exosomes were loaded with the Rhodamine Green labeled ATP aptamer through electroporation, sonication, freeze-thaw, or incubation (detailed procedures for each loading method are described in the methods section). After each method, 3 cycles of ultrafiltration with 50 kDa MWCO Amicon tubes were performed to purify the aptamer-loaded exosomes from unloaded aptamer. The aptamer-loaded exosomes were incubated with 0.1% Triton X-100 at 37°C for 10 minutes before fluorescent measurements to fully break the exosomal membrane and allow for the Rhodamine Green-labeled ATP aptamer to be released into the solution. To determine the loading efficiency, the fluorescence emission at 520 nm for each sample was fit into the standard curve best fit equation from (D). After solving for the corresponding concentration based on the standard curve, the loading efficacy was calculated as a percentage of the final ATP aptamer concentration over the initial aptamer concentration added to each sample before the loading procedures. (F) After optimizing the loading procedure, NBC-Exos were loaded with the ATP aptamer sensor (hybridized with a quencher strand). The samples were then incubated with either 2 mM ATP or 2 mM ATP + 0.1% Triton X-100 for 10 minutes at 37°C for 10 minutes to determine active sensor response. All measurements were taken in 50 mM Tris, 100 mM NaCl, 5 mM $MgCl_2$ at 37°C. Excitation at 480 nm, emission collected at 520 nm, with 5 mm slit widths.

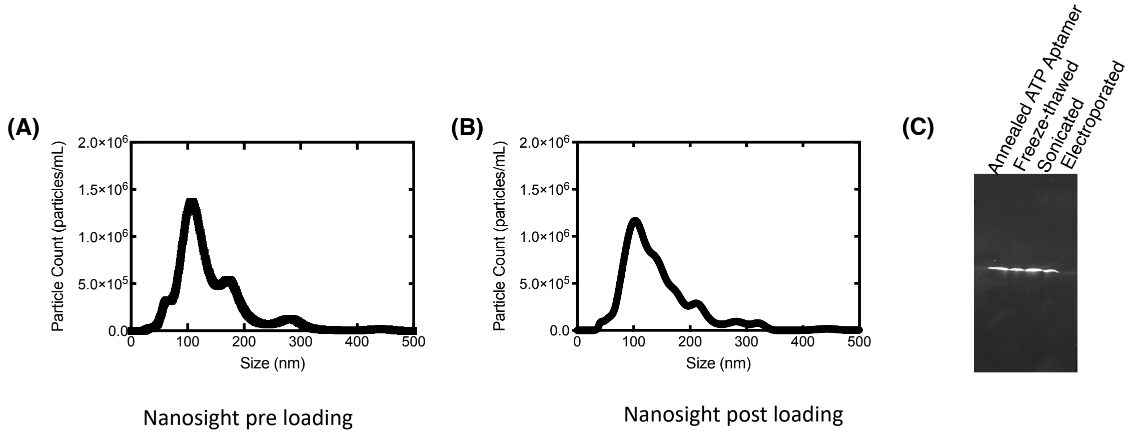

Figure S3: Evaluation of exosome and aptamer integrity pre and post loading. Exosome size, purity, and concentration were characterized by Nanosight NS500. (A) 5 µL of the resuspended exosome stock was diluted in 995 µL PBS buffer. Run times of 1 minute, with the camera level set to 8 at room temperature and screen gain set to 1 were used to collect size and purity data of unloaded exosomes. (B) Rhodamine Green-labeled ATP aptamer sensor loaded exosomes were rerun on the Nanosight using the same settings. Sensor loaded exosomes that showed slight exosome size distribution changes compared to preloaded exosomes. However, no significant distribution changes occurred, such as major aggregation. (C) Rhodamine Green-labeled aptamer integrity after loading was checked with a 10% urea denaturing PAGE gel. After each tested loading method, 10 nmol of total DNA was heated with 2X gel loading dye stop solution to 95°C for 10 minutes. The samples were loaded onto a 10% urea denaturing PAGE gel and run at 26 W for 1 hour. As each lane showed only one detectable band that corresponded to the control lane (ATP aptamer that was only incubated in buffer), we determined the loading methods did not degrade the aptamer. Detailed loaded methods are outlined in the methods section.

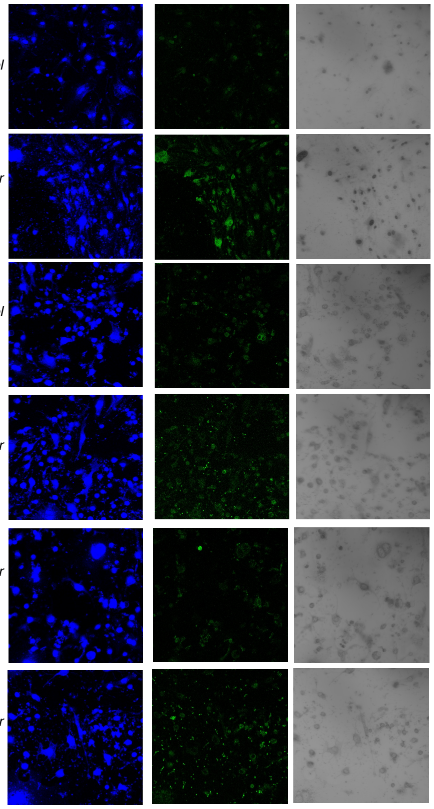

Figure S4: Monocultured bEnd.3 cell delivery confocal images. Lipofectamine 3000 or the range of native exosomes were used to deliver the Rhodamine Green-labeled ATP aptamer sensor or its scrambled inactive control into monocultured bEnd.3 cells. After a four hour delivery, cells were washed to remove background signal and nuclei were stained with Hoechst 33342. Successful aptamer delivery was visualized through the green fluorescence channel, with all active aptamer groups displaying higher fluorescence signal than their inactive scrambled control. Scale bar is 20 μM.

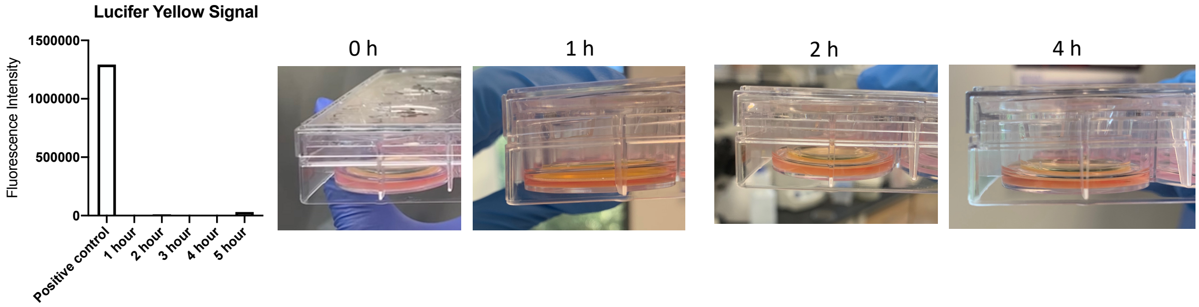

Figure S5: Transwell permeability assay. After plating the Transwell BBB cells (bEnd.3 in the apical layer and SY5Y in the basolateral layer), cells were allowed to grow for 10 days. 0.1 mg/mL Lucifer yellow was added to the apical layer and allowed to incubate in the model for 5 hours. Any Lucifer yellow signal in the basolateral cell layer represented a leaky BBB model because Lucifer yellow is not cell permeable and could only pass through gaps between bEnd.3 cells. Low signal as determined quantitatively through fluorescence intensity from 150 µL media aliquots from the SY5Y media layer. These aliquots were transferred to a 96 well plate (Corning). The fluorescence intensities of these aliquots were measured with excitation: 430 nm, emission 535 nm on a Biotek plate reader. Permeability was also qualitatively visually indicated by the yellow Lucifer Yellow dye migration from the bEnd.3 cells to the SY5Y cell media. Persistent yellow fluorescent signal in the top bEnd.3 cells media indicated the bEnd.3 cells were tightly packed even at the 5-hour time point.

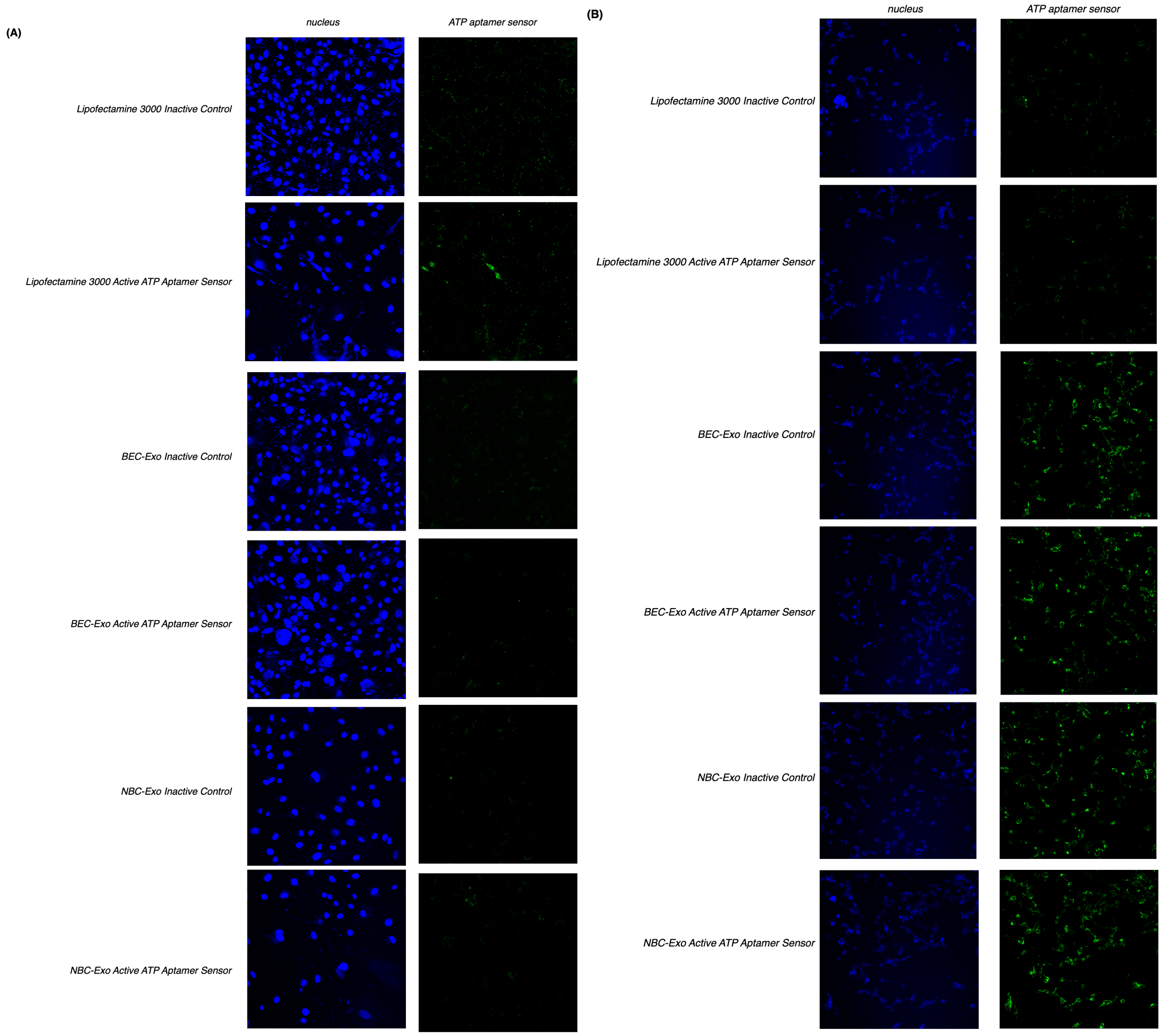

Figure S6: Five-hour incubation in Transwell BBB model with the ATP aptamer sensor. Lipofectamine 3000 or the range of native exosomes were used to deliver the Rhodamine Green-labeled ATP aptamer sensor or its scrambled inactive control into the Transwell BBB model. After a five-hour delivery, cells were washed to remove background signal and nuclei were stained with Hoechst 33342. (A) Representative images for each sample for the top bEnd.3 cell layer. (B) Representative images for each sample for the target SY5Y cell layer. Scale bar is 20 µmeter. Images could only be taken at 10X objective due to the Zeiss 710's working distance limitations and Transwell model size.

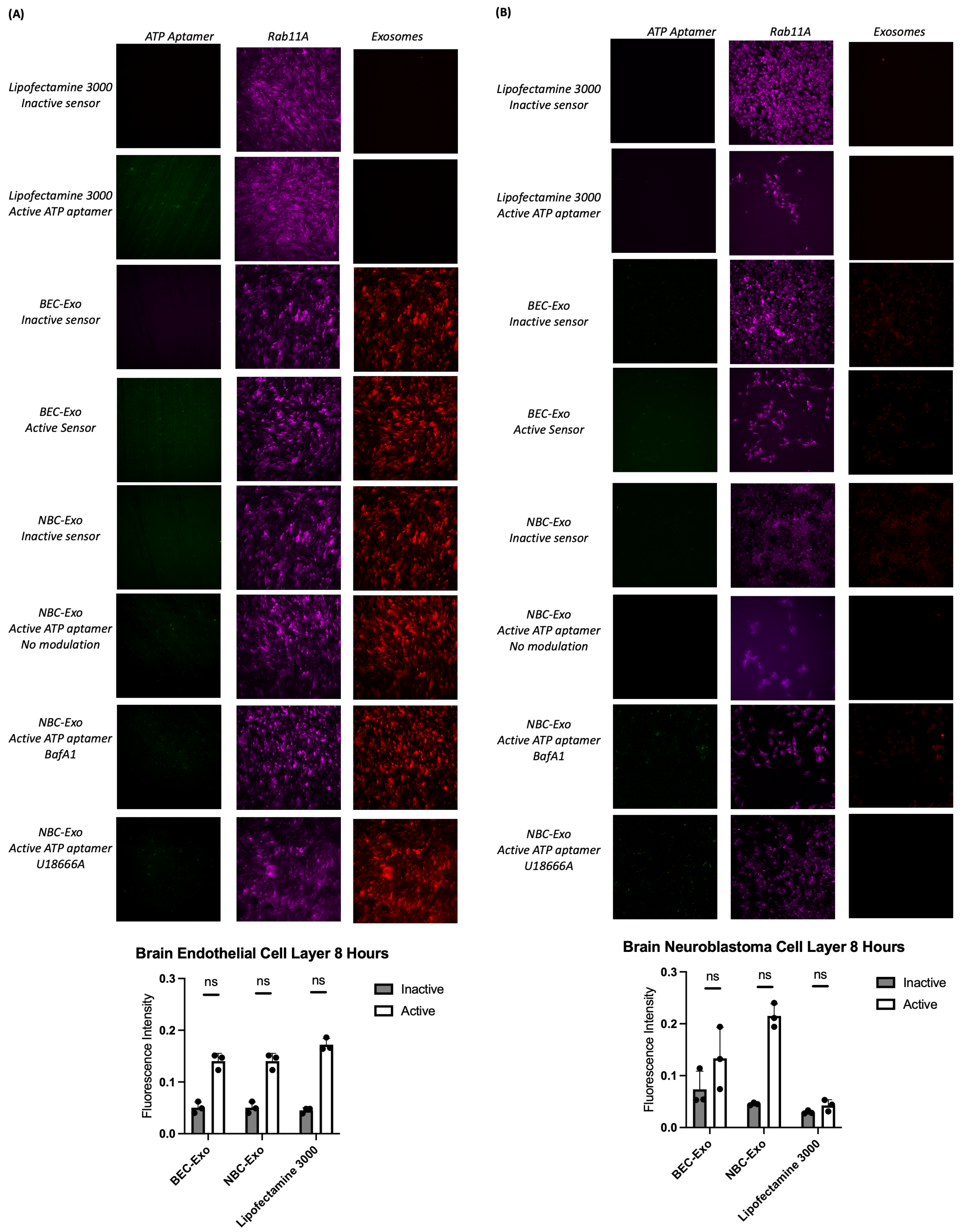

Figure S7: Eight-hour incubation in Transwell BBB model with the ATP aptamer sensor. Lipofectamine 3000 or the range of native exosomes were used to deliver the Rhodamine Green-labeled ATP aptamer sensor or its scrambled inactive control into the Transwell BBB model. After an eight-hour incubation, cells were washed to remove background signal. Cells were fixed, permeabilized, and stained with anti-Rab11A. (A) Representative images for each sample for the top bEnd.3 cell layer. (B) Representative images for each sample for the target SY5Y cell layer. Scale bar is 20 µmeter. Statistical analysis was determined through a one-way ANOVA, with data represented as means of technical replicates and error as standard deviation of the mean.

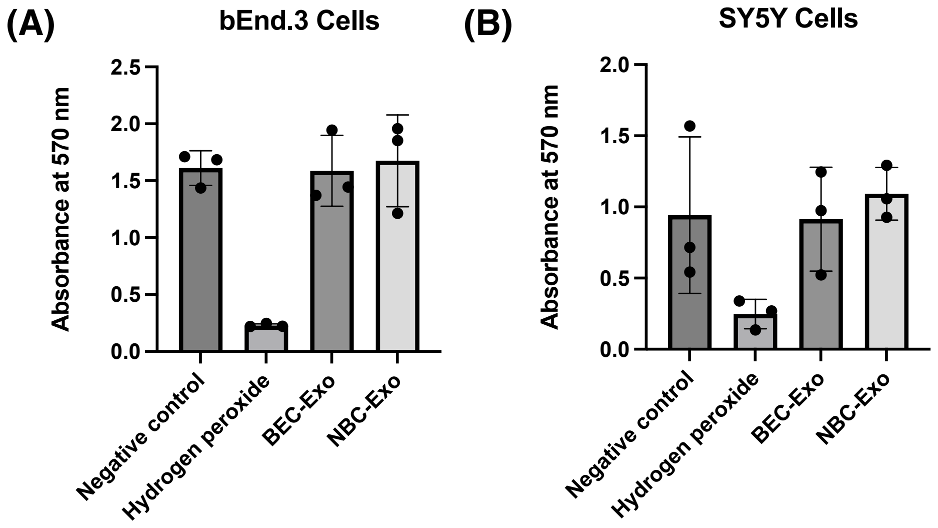

Figure S8: Cell toxicity assay for model cell types. MTT assay to determine exosomal cytotoxicity for brain endothelial cells (A) and brain neuroblastoma cells (B). Positive controls of 30% hydrogen peroxide were used to achieve maximum cell death as a comparison to a negative control of only Opti-MEM and samples of sensor-loaded exosomes. Based on the minimal change in absorbance compared to the negative control, all of the sensor-loaded exosomes displayed minimal cellular toxicity at concentrations used for in vitro cellular studies and in vivo mouse studies. Experiment was performed with three biological replicates. Error show in standard deviation of the mean.

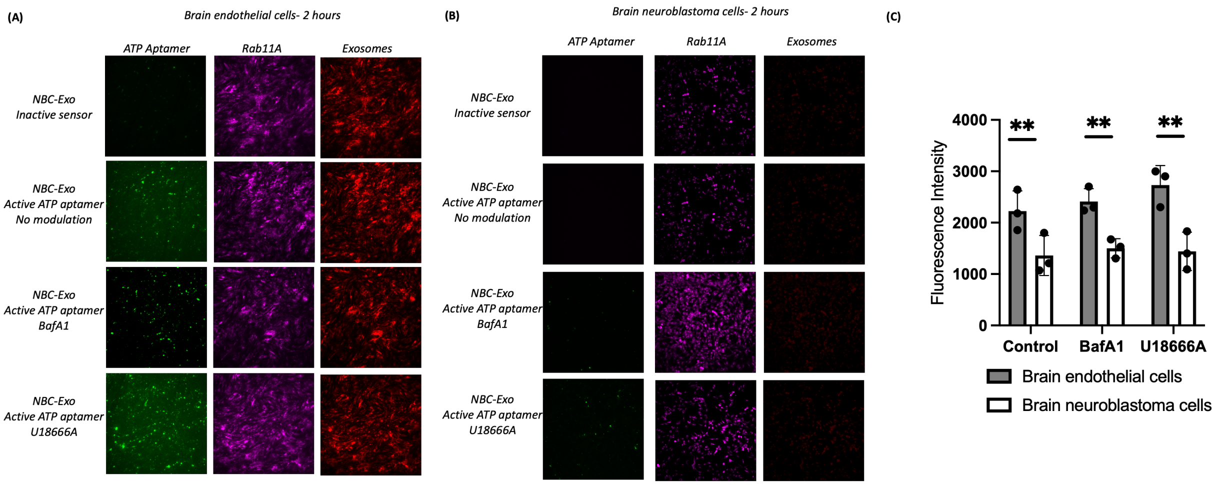

Figure S9: Two-hour incubation in Transwell BBB model with the ATP aptamer sensor for recycling endosome modulation. Lipofectamine 3000 or the range of native exosomes were used to deliver the Rhodamine Green-labeled ATP aptamer sensor or its scrambled inactive control into the Transwell BBB model. After a two-hour delivery, cells were washed to remove background signal. Cells were fixed, permeabilized, and stained with anti-Rab11A for simultaneous evaluation with the recycling endosome pathway. (A) Representative images for each sample for the top bEnd.3 cell layer. (B) Representative images for each sample for the target SY5Y cell layer. (C) Quantified intracellular fluorescence intensity. Scale bar is 20 µmeter. Statistical analysis was determined through a one-way ANOVA between groups, with data represented as means of technical replicates and error as standard deviation of the mean.

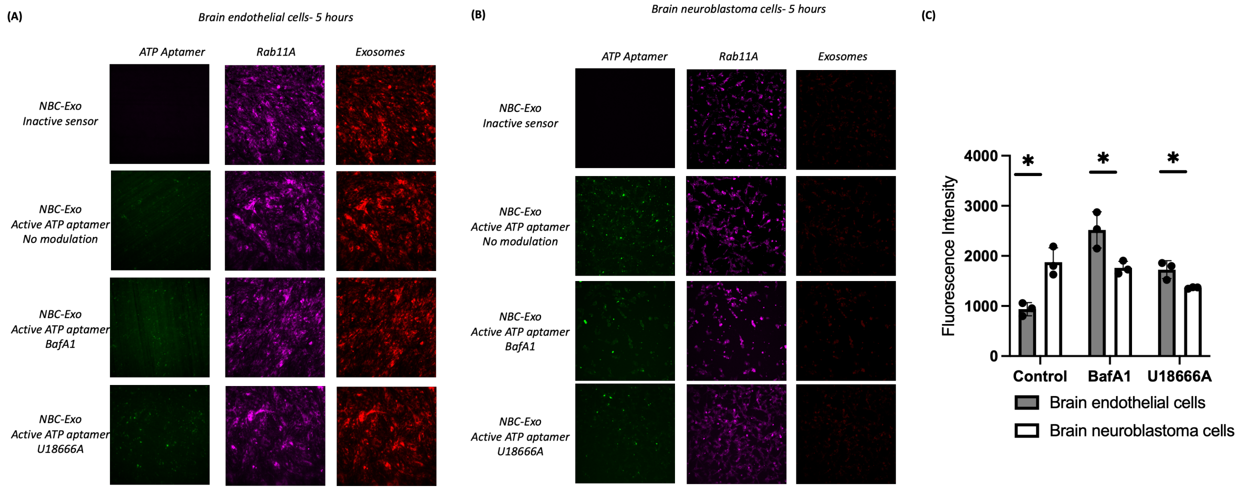

Figure S10: Five-hour incubation in Transwell BBB model with the ATP aptamer sensor for recycling endosome modulation. Lipofectamine 3000 or the range of native exosomes were used to deliver the Rhodamine Green-labeled ATP aptamer sensor or its scrambled inactive control into the Transwell BBB model. After a five-hour delivery, cells were washed to remove background signal. Cells were fixed, permeabilized, and stained with anti-Rab11A for simultaneous evaluation with the recycling endosome pathway. (A) Representative images for each sample for the top bEnd.3 cell layer. (B) Representative images for each sample for the target SY5Y cell layer. (C) Quantified intracellular fluorescence intensity. Scale bar is 20 µmeter. Statistical analysis was determined through a one-way ANOVA between groups, with data represented as means of technical replicates and error as standard deviation of the mean.

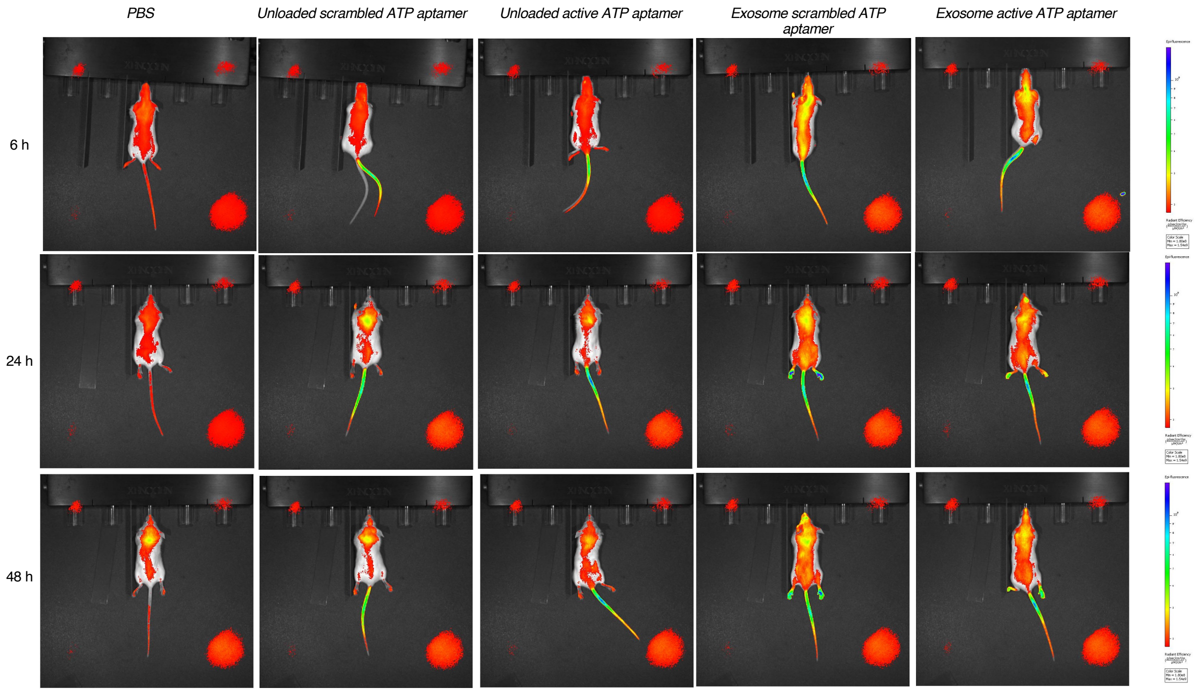

Figure S11: Live mice ATP aptamer sensor-exosome tracking. Following the general protocol for exosome loading and tail vein injections of mice, in vivo imaging was performed to evaluate the circulation of the Cy5-labeled ATP aptamer sensor over 48 hours post tail vein injection. As a negative control to account for bloodstream autofluorescence, we used PBS injections. As a negative control to account for Cy5-DNA remaining in the bloodstream, we injected the unloaded hybridized scrambled control and the unloaded active ATP aptamer sensor. For the positive samples to account for brain delivery, we injected the exosome loaded hybridized scrambled control and the exosome loaded hybridized active ATP aptamer sensor. Image parameters used were 640 nm excitation, 680 nm emission, f number 4, binning factor 8, and 6.6 field of view. Notably, exosome samples contained persistent fluorescent signal over negative controls of unloaded sensor even at the 48 hour time point. Likewise, slight central nervous system signal accumulation was observed with the exosome-mediated samples.

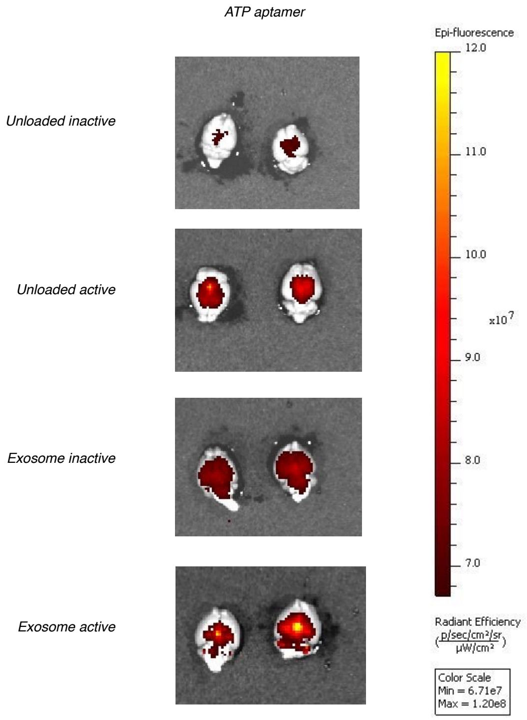

Figure S12: Whole brain IVIS imaging of the ATP aptamer sensor in 5xFAD mice. To determine how ATP changes in Alzheimer's disease, we carried out the same delivery studies across the BBB in 5xFAD mice. Following the general exosome sample preparation and tail vein injection protocols, mice were sacrificed using CO_2 and cervical dislocation. The brains were washed in 3X with PBS in a petri dish. Within 1 hour of excision, the brains were placed on the IVIS instrument and imaged using 640 nm excitation, 680 nm emission, f number 4, binning factor 8, and 6.6 field of view. Two biological replicates were performed for each sensor and experimental sample. Minimal background signal is observed in the unloaded controls due to unwashed sensor remaining in the bloodstream. Exosome sensors display higher signal intensity due to signal sequestered within the brain. Compared to the healthy BALB/c mice, the negative controls displayed higher background fluorescence. This may be due to the weaker BBB structure in neurodegenerative diseases.

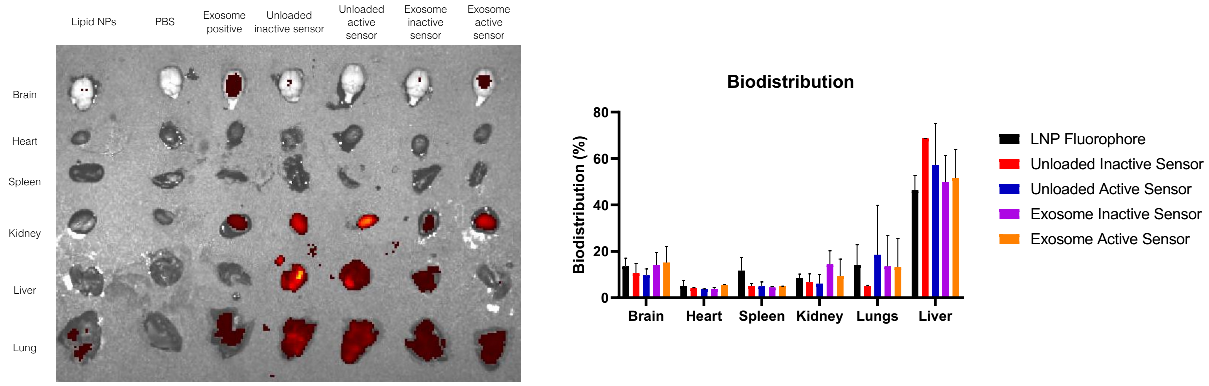

Figure S13: Biodistribution assay. To determine the delivery efficiency in vivo, we excised all major organs 6 hours post-injection and evaluated their fluorescence intensities in the IVIS. Following the general exosome sample preparation and tail vein injection protocols in BALB/c mice, mice were sacrificed using CO_2 and cervical dislocation. All major organs (brain, heart, spleen, kidney, liver, and lungs) were harvested 6 hours post tail vein injection with all control and active groups. Organs were thoroughly washed with 3X PBS in a petri dish to remove all residual blood and placed onto the IVIS device. Image parameters used were 640 nm excitation, 680 nm emission, f number 4, binning factor 8, and 6.6 field of view. Biodistribution was performed with 3 biological replicates, error is standard deviation of the mean of the three separate biological replicates. The kidney, liver, and lungs sequestered most of the fluorescence signals, whether from the lipid nanoparticle control, unloaded sensor controls, or exosome mediated controls. Brain signals were 2% higher for the exosome mediated groups compared to the unloaded controls. Interestingly, the lipid nanoparticles displayed negligible total fluorescence in all organs compared to the unloaded controls and exosome samples. Perhaps, the lipid nanoparticle group had higher body clearance and was already removed from the mouse circulation and organs at this time point.

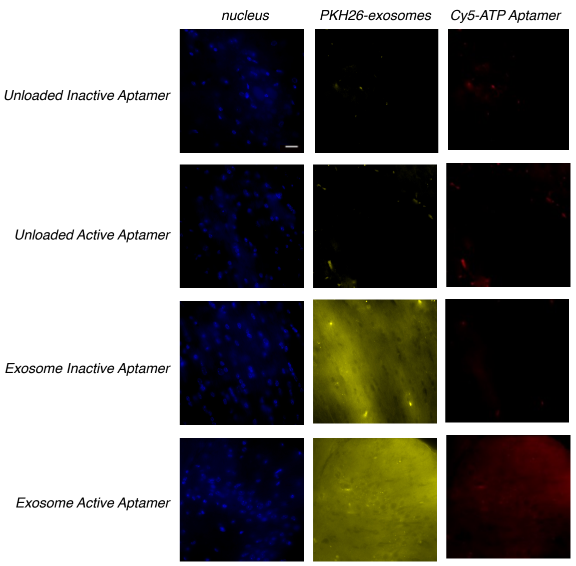

Figure S14: High resolution imaging of the ATP aptamer in the subiculum in freshly frozen brain slices after tail vein injections into BALB/c mice. Evaluating the exosome delivery locations, sensor output compared to the inactive control, and exosome and sensor colocalization with the nucleus. Scale bars are 20 μmeter.

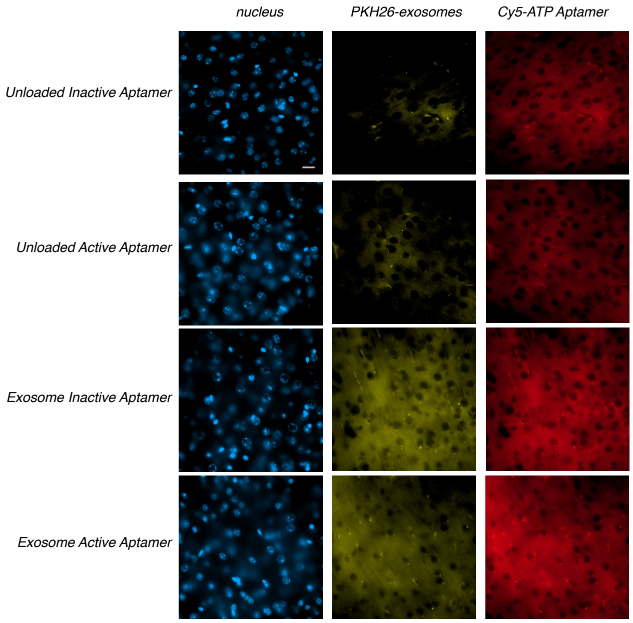

Figure S15: High resolution imaging of the ATP aptamer in the cortex in freshly frozen brain slices after tail vein injections into BALB/c mice. Evaluating the exosome delivery locations, sensor output compared to the inactive control, and exosome and sensor colocalization with the nucleus. Scale bars are 20 µmeter.

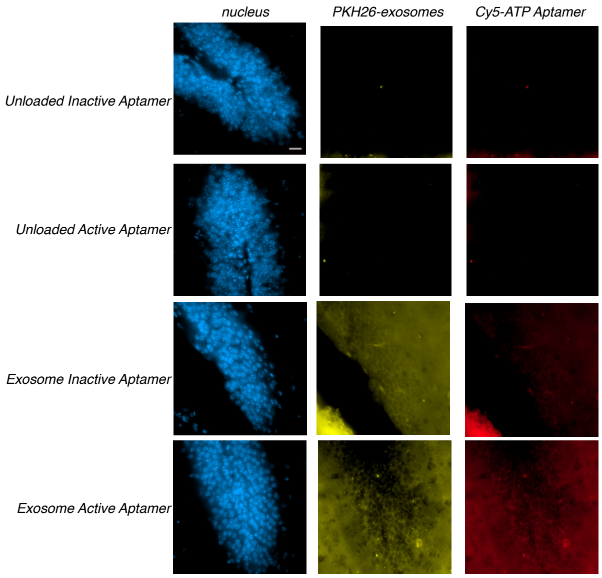

Figure S16: High resolution imaging of the ATP aptamer in the hippocampus in freshly frozen brain slices after tail vein injections into BALB/c mice. Evaluating the exosome delivery locations, sensor output compared to the inactive control, and exosome and sensor colocalization with the nucleus. Scale bars are 20 μmeter.

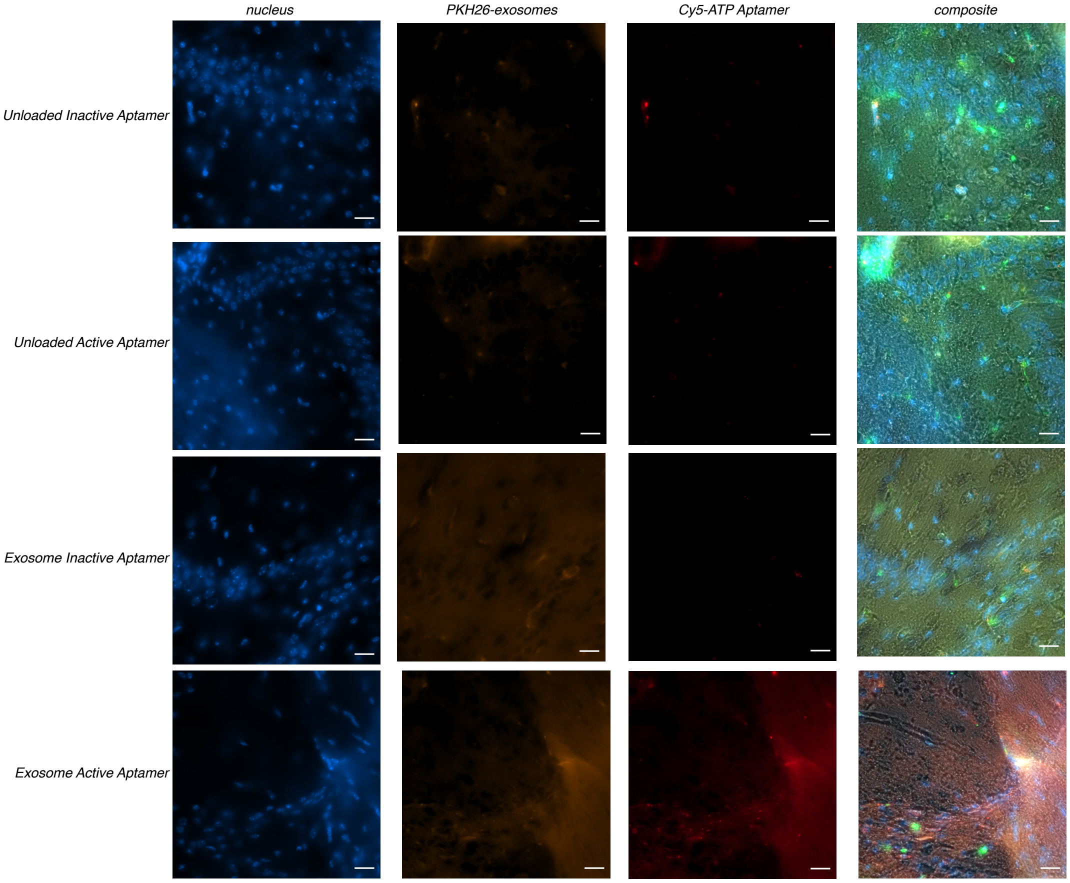

Figure S17: High resolution imaging of the ATP aptamer in the subiculum in freshly frozen brain slices after tail vein injections into 5X FAD mice. Evaluating the exosome delivery locations, sensor output compared to the inactive control, and exosome and sensor colocalization with the nucleus. Scale bars are 20 µmeter.

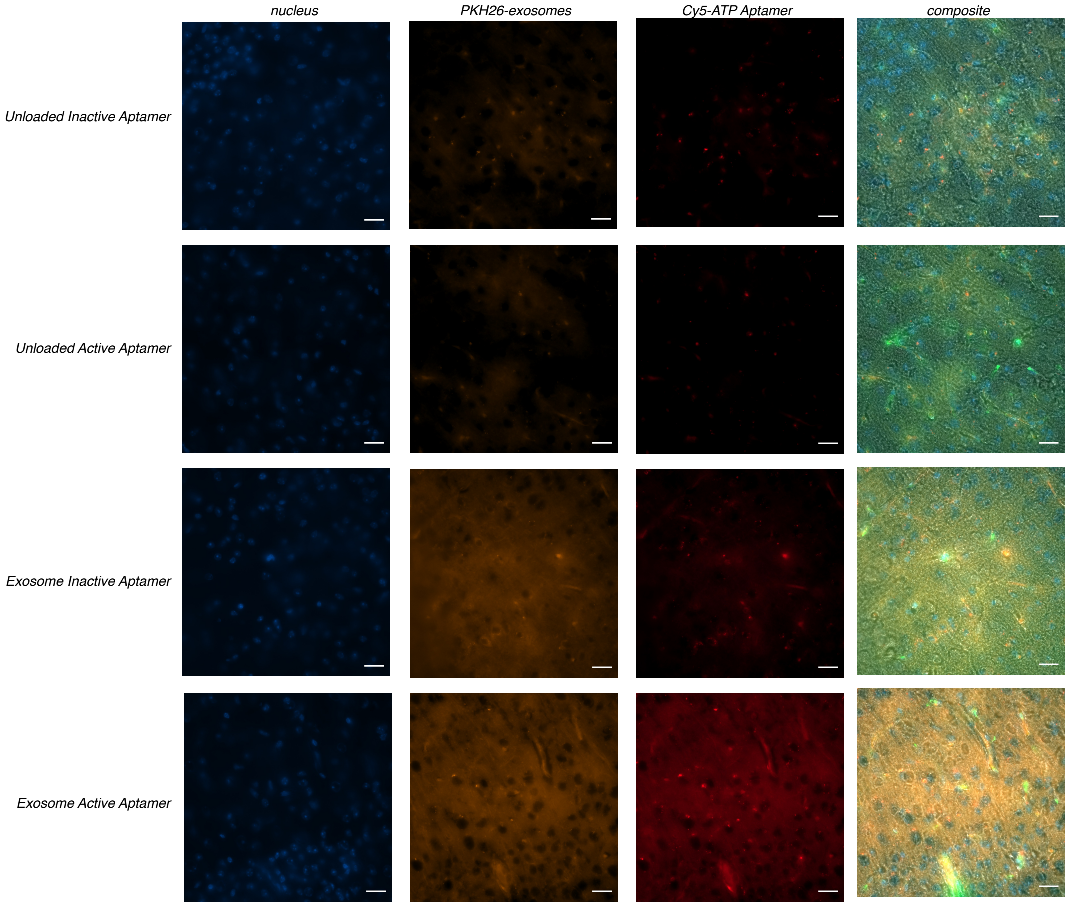

Figure S18: High resolution imaging of the ATP aptamer in the cortex in freshly frozen brain slices after tail vein injections into 5X FAD mice. Evaluating the exosome delivery locations, sensor output compared to the inactive control, and exosome and sensor colocalization with the nucleus. Scale bars are 20 µmeter.

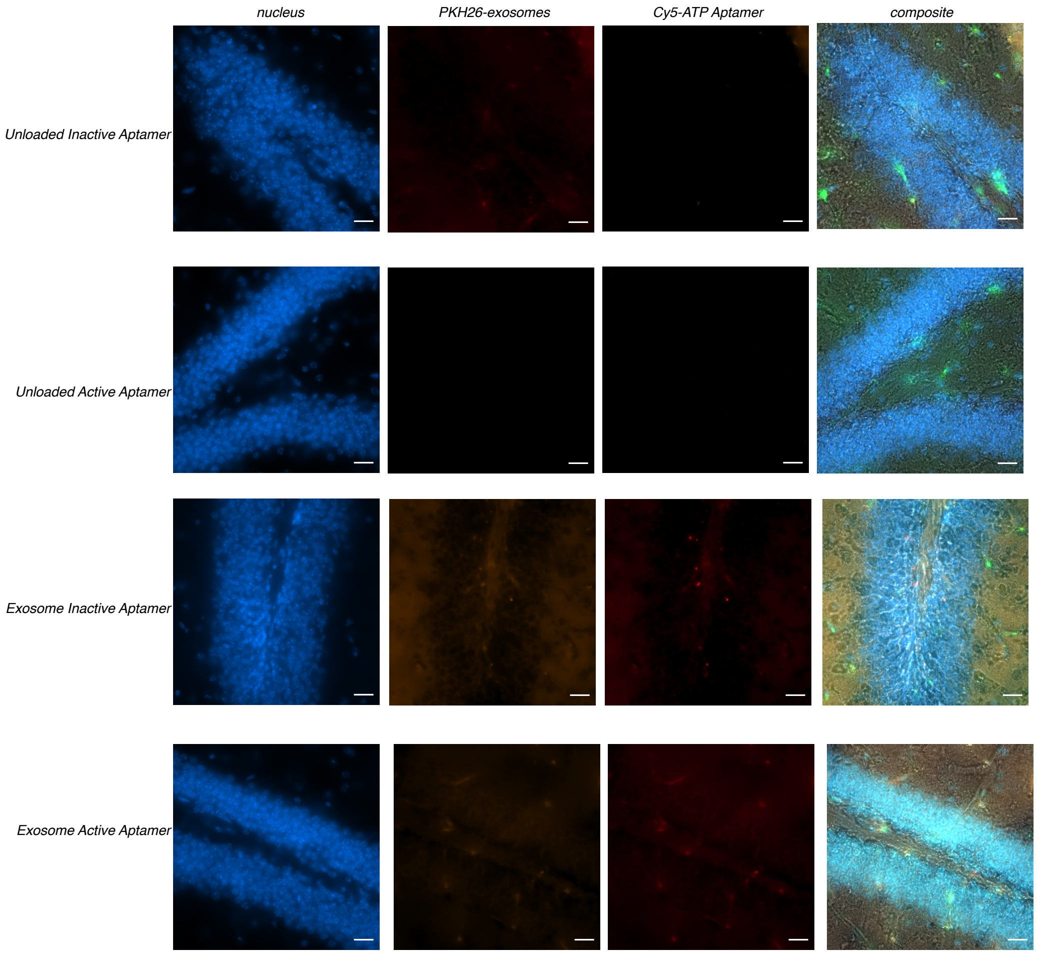

Figure S19: High resolution imaging of the ATP aptamer in the hippocampus in freshly frozen brain slices after tail vein injections into 5X FAD mice. Evaluating the exosome delivery locations, sensor output compared to the inactive control, and exosome and sensor colocalization with the nucleus. Scale bars are 20 µmeter.

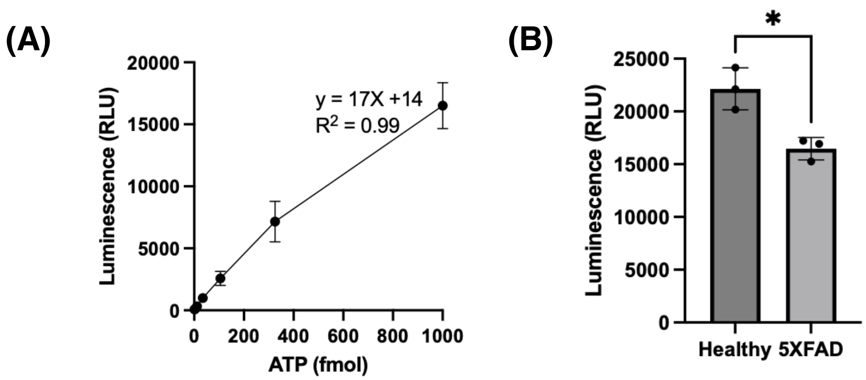

Figure S20: Positive control of a secondary method to evaluate whole brain ATP levels. (A) Standard curve of ATP concentrations in vitro, as measured by luciferase-luciferin pairs. (B) Luminescence measurements of a healthy mouse brain compared to an Alzheimer disease model 5xFAD mouse brain. When accounting for the sample volume and converting these luminescence measurements to molarity, we found the healthy brain ATP concentration was 2.02 mM and the 5xFAD brain ATP concentration was 1.53 mM. Experiment was performed in technical triplicates, with error bars representing standard deviation. Statistical analysis is an unpaired t-test, with $p^* < 0.05$.
